# Evolutionary analyses of the animal glycosyltransferase family 54 reveals two β1,4-N-acetylglucosaminyltransferase families

**DOI:** 10.1016/j.isci.2025.113788

**Published:** 2025-10-15

**Authors:** Aoi Morigo, Roxana Elin Teppa, Masamichi Nagae, Hirokazu Yagi, Yasuhiko Kizuka, Anne Harduin-Lepers

**Affiliations:** 1 Graduate School of Natural Science and Technology, Gifu University, Gifu 501-1193, Japan; 2 Univ. Lille, CNRS, UMR 8576 - UGSF - Unité de Glycobiologie Structurale et Fonctionnelle, 59000 Lille, France; 3 Department of Molecular Immunology, Research Institute for Microbial Diseases, Osaka University, Suita 565-0871, Japan; 4 Laboratory of Molecular Immunology, Immunology Frontier Research Center (IFReC), Osaka University, Suita 565-0871, Japan; 5 Graduate School of Pharmaceutical Sciences, Nagoya City University, Nagoya 467-8603, Japan; 6 Institute for Glyco-core Research (iGCORE), Gifu University, Gifu 501-1193, Japan

**Keywords:** Biochemistry, Evolutionary biology

## Abstract

*N*-acetylglucosaminyltransferases involved in branched *N*-glycans synthesis, a major post-translational modification, are gathered in the CAZy glycosyltransferase family 54. To date, the origin and evolution of this biosynthetic pathway are unknown, and the functional organization of the Golgi enzymes remains elusive. Over 230 metazoan GT54-related genes were identified, and sequence-based analysis of vertebrate MGAT4 proteins shed light on evolutionary conserved peptide motifs and structural features like the lectin domain (CBM94). Molecular phylogeny analyses disentangled their evolutionary relationships, revealing the deep ancestry of two metazoan clusters, and unveiled the existence of seven vertebrate MGAT4 subfamilies. Comparative genomics and sequence-based analyses identified an evolutionarily conserved subgroup of GT54 gathering MGAT4A, MGAT4B, and MGAT4D, whereas the other subgroup comprised of MGAT4C, MGAT4E, MGAT4F, and MGAT4G evolved faster. Biochemical analyses conducted with representatives of each subgroup revealed the existence of two acceptor substrate specificities and suggested their intricate functional organization with the other Golgi branching enzymes.

## Highlights

• Molecular phylogeny disentangles evolutionary relationships of 7 vertebrate MGAT4 subfamilies
• Two MGAT4 families with deep ancestry emerged in Metazoa
• Sequence-based analyses reveal conserved structural features in each MGAT4 family
• Functional genomics identifies two acceptor substrate specificities in each MGAT4 family

## Graphical abstract

**Figure:**
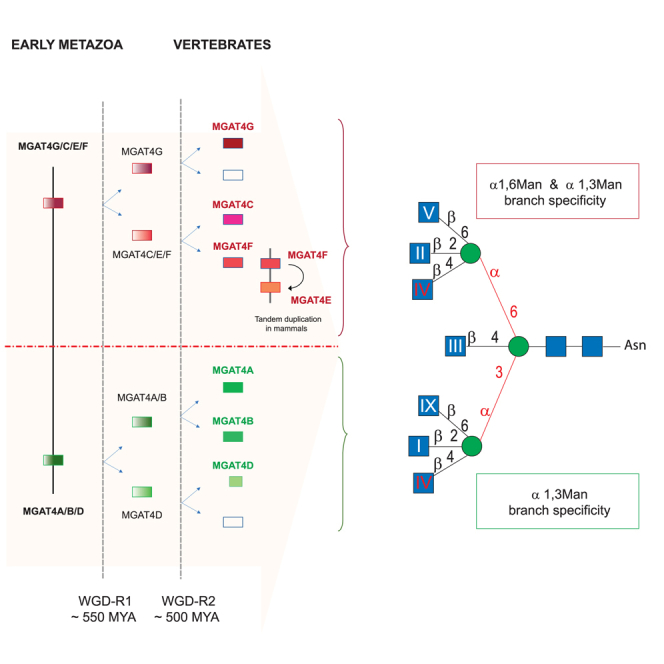

## Introduction

*N*-glycosylation of proteins is one of the most frequent and universal post-translational modifications (PTMs)^1^ promoting proteostasis in the secretory pathway. The largest diversity of *N*-glycan structures ranging from paucimannosidic to di-, tri-, tetra-, and penta-antennary *N*-glycans has been described in Bilateria,^2^ although penta-antennary *N*-glycans are not present in human tissues. One major factor conferring structural variation of *N*-glycans is the variable number of GlcNAc branches,^3^ which regulates the function of proteins like molecular interactions with carbohydrate-binding proteins.^4^^,^^5^^,^^6^ Branched *N*-glycans are associated with various biological functions of cell adhesion molecules (e.g., E-cadherin and integrins) and cell surface receptors, including cell adhesion and cancer metastasis, and metabolic pathways such as type II diabetes.^7^^,^^8^ It is therefore pivotal to understand the origin and evolution of this branched *N*-glycan biosynthetic pathway and how the structural diversity is generated in cells.

**Figure 1:**
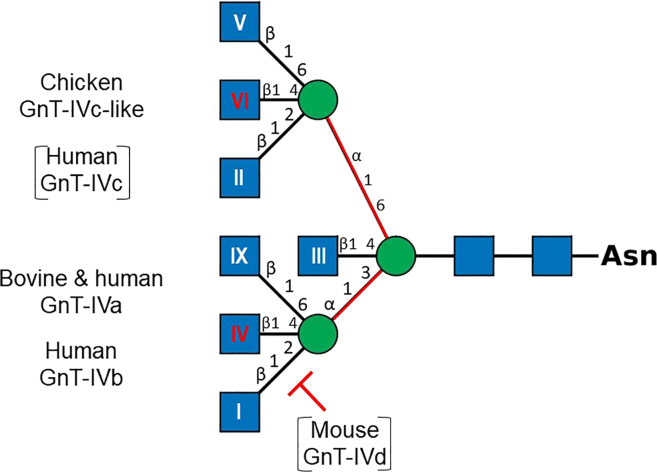
Schematics representing structure of *N*-glycan branches produced by MGAT4 enzymes The *N*-glycan structure is represented according to the symbol nomenclature for glycans (SNFG) where blue squares and green circles represent GlcNAc and Man residues, respectively (https://www.ncbi.nlm.nih.gov/glycans/snfg.html).^26^ Roman numbers in the GlcNAc symbol indicate the name of GlcNAc transferases previously shown to synthesize the indicated GlcNAc branches, i.e., I: GnT-I (MGAT1); II: GnT-II (MGAT2); III: GnT-III (MGAT3); IV: GnT-IV (MGAT4); V: GnT-V (MGAT5); VI: GnT-VI/GnT-IV (MGAT6/MGAT4), and IX: GnT-IX (MGAT5B). The vertebrate MGAT4s that have been cloned and characterized are indicated. Recombinant bovine and human MGAT4A and human MGAT4B act on α1,3-Man arm to produce the tri-antennary *N-*glycan, while the chicken MGAT4C-like was shown to act after MGAT5 on α1,6-Man arm to produce the tetra-antennary *N*-glycan and was designated as MGAT6. The α1,3-Man and α1,6-Man linkages are indicated in red. Recombinant human MGAT4C and mouse MGAT4D showed no transferase activity but mouse MGAT4D inhibited the MGAT1 activity and are indicated in brackets.

In the eukaryotic *N*-glycosylation pathway, the enzyme machinery leading to oligosaccharide structures formed in the endoplasmic reticulum (ER) and transferred onto nascent proteins is remarkably conserved, whereas the Golgi terminal glycosylation machinery is highly diverse and confers huge protein functional modularity.^9^^,^^10^^,^^11^ The variable number of GlcNAc branches is acquired during *N*-glycan maturation in the Golgi.^3^^,^^12^ After *N*-glycosylated proteins enter the *cis*-Golgi, the terminal mannose (Man) residues are trimmed by mannosidases, and the β1,2-*N*-acetylglucosaminyltransferase 1 (GnT-I, MGAT1) transfers *N*-acetylglucosamine (GlcNAc) to the α1,3Man, initiating the biosynthesis of hybrid and complex-type *N*-glycans. After further Man trimming by MAN2A1 and MAN2A2, the β1,2-*N*-acetylglucosaminyltransferase 2 (GnT-II, MGAT2) adds GlcNAc to form bi-antennary *N*-glycans. The medial Golgi MGAT1 and MGAT2 show relatively high affinity (i.e., ∼0.04 and 0.9 mM, respectively) for their donor substrate UDP-GlcNAc supplied by the hexosamine pathway from glucose, glutamine, and acetyl-coenzyme A,^13^ leading to the efficient synthesis of a bi-antennary *N*-glycan. This *N*-glycan is a substrate for various glycosyltransferases (GTs), including GlcNAc-, galactose- (Gal), *N*-acetylgalactosamine- (GalNAc), and sialyltransferases. Several vertebrate enzymes are essential in producing multi-antennary sugar chains, which are absent in plants and fungal *N*-glycans.^14^ As illustrated in Figure 1, these enzymes include the β1,4-mannosyl-glycoprotein β1,4-*N*-acetylglucosaminyltransferase MGAT3 (GnT-III),^15^ the α1,3-mannosyl-glycoprotein β1,4-*N*-acetylglucosaminyltransferases MGAT4A (GnT-IVa), MGAT4B (GnT-IVb), MGAT4C (GnT-IVc, GnT-VI, or GnT-IV-H), and MGAT4D (GnT-1IP-L),^8^^,^^16^^,^^17^^,^^18^^,^^19^^,^^20^ and the α1,6-mannosyl-glycoprotein β1,6-*N*-acetylglucosaminyltransferases MGAT5 (GnT-V)^21^^,^^22^ and MGAT5B (GnT-IX or GnT-Vb),^23^ which further increase the number of branches. MGAT4A and MGAT5 show much lower affinity to UDP-GlcNAc compared to MGAT1 and MGAT2 (i.e., ∼5 and 11 mM, respectively),^24^ which further suggests a switch-like activation of these branching enzymes with increasing concentration of UDP-GlcNAc, leading to the formation of tri- and tetra-antennary *N*-glycans.^13^^,^^25^

The formation of branched *N*-glycans is vital for protein functions,^27^ yet our current understanding of the functional organization of this glycosylation machinery remains fragmentary. To gain insights into the biological function of the branched *N*-glycans, the human enzymes MGAT4A and MGAT4B were cloned and characterized.^28^^,^^29^ Mammalian enzymes were found to transfer GlcNAc residues from UDP-GlcNAc to the α1,3-Man arm of an *N*-glycan via a β1,4-linkage (GnT-IV activity) *in vitro.*^30^^,^^31^ MGAT4C was cloned from human^32^ and chicken^19^ tissues and MGAT4D from mouse tissues,^33^ but no GlcNAc transfer activity could be established for human MGAT4C and mouse MGAT4D. However, another function was shown for MGAT4D, which acts as an inhibitor of MGAT1 via its luminal domain suppressing *N*-glycan conversion from oligomannose to complex type glycans.^16^^,^^33^ Intriguingly, a chicken MGAT4C-like enzyme showed the ability to transfer a GlcNAc residue on the α1,6-Man arm of a tri-antennary *N*-glycan and was once named GnT-VI (MGAT6).^19^^,^^34^^,^^35^^,^^36^ Ectopic expression of this bird GnT-VI activity, notably absent in mammals, together with the GnT-V in human cultured cells formed a β1,4 linkage, allowing conversion of tri- and tetra-branched to tetra- and penta-branched *N*-glycans that regulated galectin binding to growth factor receptors and nutrient transporters at the cell surface.^13^^,^^37^ Besides the biological relevance of these branching enzymes, these studies raised the possibility of the existence of a divergent subset of MGAT4 enzymes in vertebrates and uncovered the need for a clear nomenclature of GT54-related CAZymes.

In the Carbohydrate-Active Enzyme (CAZy) classification,^38^ the *N*-glycan branching enzymes fall into five distinct CAZy families: GT13 for MGAT1-, GT16 for MGAT2-, GT17 for MGAT3-, GT18 for MGAT5-, and GT54 for the MGAT4-related proteins. The human MGAT5 (GT18) was shown to adopt a GT-B fold, consisting of two Rossmann folds,^39^ whereas GT13 and GT16 adopt a GT-A fold. GT17 and GT54 were proposed to adopt a GT-A fold,^40^^,^^41^ and in the evolutionary analysis of fold A GTs (GT-A) of Taujale et al., the GT54 and GT17 proteins are placed in a separate clade.^42^^,^^43^ The 3-dimensional structure and function relationships of the MGAT4 proteins and mechanisms regulating their activity are not yet elucidated. MGAT4 proteins possess multiple domains, and recently, a lectin domain corresponding to the carbohydrate-binding module 94 (CBM94) was identified in the C-terminal region of mouse MGAT4A^44^; the crystal structures of the lectin domain of the human MGAT4A (PDB 7XTL) and of the silkworm *Bombyx mori* (PDB 7XTM) were determined.^45^ Although the function of this domain remains unclear, it has been proposed to influence glycoprotein substrate preference and self-regulation of MGAT4.^8^^,^^31^^,^^44^^,^^46^ Remarkably, this lectin domain is not found in the human MGAT4D.^8^ From an evolutionary perspective, the origin and fate of the MGAT4 genes remain an enigma.^38^^,^^39^^,^^40^^,^^41^^,^^42^^,^^43^^,^^44^ Taken together, these data suggest distinct structure-function and evolutionary relationships of the branching enzymes.

To trace the evolutionary relationships of the GT54 and enhance our understanding of the molecular mechanism of the human enzymes, we identified over 230 GT54-related sequences in multicellular animals. Combining bioinformatics and biochemical approaches, we unveiled the existence of three additional vertebrate GT54 subfamilies named MGAT4E, MGAT4F, and MGAT4G. Our comprehensive phylogenetic analyses revealed that the GT54 family is split into two deeply rooted branches in metazoans and seven clades in vertebrates. We also conducted functional assays with mouse GnT-IVa-GnT-IVf, chicken GnT-IVc and GnT-IVf, and medaka GnT-IVc and GnT-IVd and showed that in the first GT54 subgroup, MGAT4A, MGAT4B, and MGAT4D are α1,3-mannosyl-glycoprotein β1,4-*N*-acetylglucosaminyltransferases, whereas in the second GT54 subgroup, MGAT4C, MGAT4E, MGAT4F, and MGAT4G are α1,6-mannosyl-glycoprotein β1,4-*N*-acetylglucosaminyltransferases acting on tri-antennary *N*-glycans. Collectively, our data suggest that GT54 is a genomic novelty of metazoans and that they clarify the evolutionary relationships of the seven vertebrate MGAT4 subfamilies shedding lights into the functional divergence of vertebrate GnT-IV enzymes.

## Results and discussion

### Existence of two deeply rooted GT54 subgroups and seven vertebrate subfamilies

#### GT54 genes are restricted to Metazoa

To initiate the search of GT54-related sequences, the human GT54 MGAT4A, MGAT4B, MGAT4C, and MGAT4D^8^ were chosen as representative members for the search input. This survey yielded hundreds of potential GT54 homologues from a diverse array of 103 metazoan genomes (Table S1 in Data S1).^47^ They appeared at the onset of Metazoa with several copies present in the early branching cnidaria *Nematostella vectensis*, *Exaiptasia diaphana*, *Pocillopora damicornis*, and *Actinia tenebrosa*, whereas other early emerging metazoan branches like Ctenophora lack GT54-related sequences, further suggesting that some metazoan branches lost MGAT4-related genes during evolution. Nonmetazoan outgroups like Choanoflagellata, the closest Metazoa sister group of extant unicellular relatives of metazoans,^47^ or other multicellular organisms with multiple origin like Fungi and Viridiplantae, or Amobozoa, the sister branch of Opisthokonta, were also investigated, but no MGAT4-related sequence could be identified. These later observations corroborate the fact that no MGAT4 branched *N*-glycan structures are described in the Amoebozoa *Dictyostelium discoideum*,^48^ further suggesting that GT54 could be a metazoan novelty.^49^ In addition, early branching vertebrates like teleosts and amphibians appear to have an expanded and very diverse GT54 repertoire, which is lost in mammals (Table 1). It was desirable to gain insights into the GT54 evolutionary history that correctly described the distribution of the GT54 genes in Metazoa. Although their evolutionary origin is not yet resolved, their distribution resembles that of other *N*-glycan branching enzymes like GT18 (MGAT5) and GT17 (MGAT3) and differs from the one of later-acting Golgi enzymes like the widespread sialyltransferases in the main eukaryotic branches and the β1,3-glycosyltransferases (GT29 and GT31) found in early eukaryotes.^10^^,^^50^^,^^51^ To establish their evolutionary trajectories and reliably classify them, the identified GT54-related sequences were further studied according to four complementary criteria: (1) gene structure, (2) sequence/structure similarities, (3) molecular phylogeny, and (4) sequence similarity network.

**Table 1:** Distribution of GT54 in Metazoa

Ancestral (before 2 WGDR)	Ohnologues (after 2nd WGDR)	Other names	Fish (after 3rd WGDR)	Fish (after 4th WGDR)	Amphibians	Birds + snakes	Mammals
**GR1: MGAT4A, MGAT4B, MGAT4D**							

MGAT4A/B/D	MGAT4A	–	MGAT4A	MGAT4A-rA	MGAT4A	MGAT4A	MGAT4A
		–		MGAT4A-rB			
	MGAT4B	–	MGAT4B	MGAT4B-rA	MGAT4B	MGAT4B	MGAT4B
		–		MGAT4B-rB			
	MGAT4D	MGAT4B-like in fish GnT1IP-L in human	MGAT4D	MGAT4D-rA	MGAT4D	MGAT4D	MGAT4D CBM94 lost
				MGAT4D-rB			

**GR2: MGAT4C, MGAT4E, MGAT4F, MGAT4G**							

MGAT4C/F/G	MGAT4C	hGnT-IV-H	MGAT4C	MGAT4C-rA	MGAT4C	MGAT4C	MGAT4C
				MGAT4C-rB			
	–	–	–	–	–	–	MGAT4E MGAT4EP in human
	MGAT4F	MGAT4C-like or MGAT6 in birds	LOST except in gar	MGAT4F	LOST	MGAT4F	MGAT4F MGAT4FP in human
	MGAT4G	–	LOST	LOST	MGAT4G	MGAT4G	LOST

This table shows the two orthologue groups of GT54 found in metazoan genomes and the seven vertebrate subfamilies. Newly described vertebrate subfamilies include MGAT4E, MGAT4F, and MGAT4G. WGDR, whole-genome duplication round.

#### Gene structure analyses identify two GT54 subgroups

**Figure 2:**
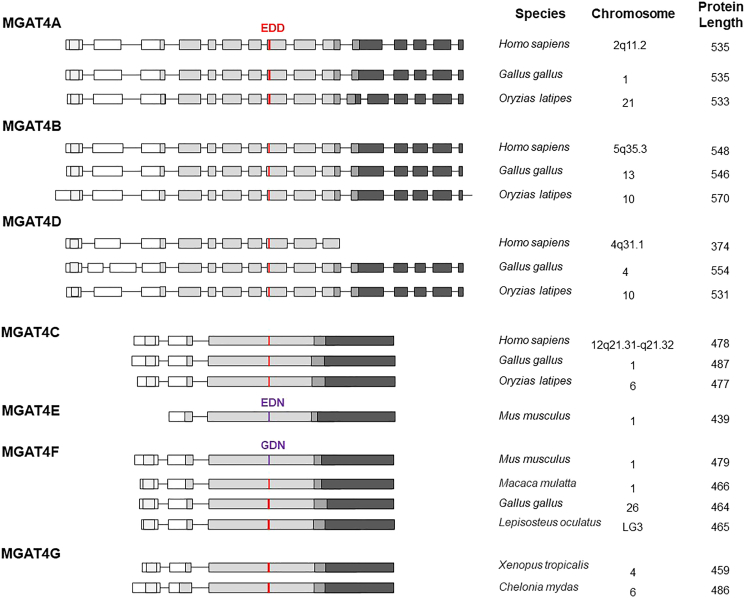
Genomic organization of the MGAT4 gene family in vertebrates Representative MGAT4 genes from different vertebrate branches including fish (*Oryzias latipes* or *Lepisosteus oculatus*), amphibians (*Xenopus tropicalis*), sauropsidae (*Gallus gallus* or *Chelonia mydas*), and mammals (*Homo sapiens*, *Mus musculus*, or *Macaca mulata*) are schematized. These genes are polyexonic, and coding exons are represented as rectangles. Vertebrate MGAT4A, MGAT4B, and MGAT4D genes show 14 or 15 translated exons with conserved exon/intron boundaries with the exception of the mammalian MGAT4D genes, which only have 10 coding exons. The MGAT4C, MGAT4E, MGAT4F, and MGAT4G genes present another gene organization with only two or three coding exons. Corresponding protein features are indicated: light gray boxes represent transmembrane region, middle gray boxes delineate catalytic domain, dark gray boxes show the linker region, and black boxes represent the lectin domain, CBM94. The conserved DxD motifs (EDD) are shown in red, whereas the mutated DxD motifs (EDN and GDN) in mouse MGAT4E and MGAT4F are shown in purple. Chromosomal location and amino acid length of enzyme are indicated on the right.

The gene organization analysis of the coding region of vertebrate-MGAT4-related genes revealed the existence of two sets of multiexonic genes, which are distributed across different vertebrate chromosomes (Figure 2). The MGAT4A, MGAT4B, and MGAT4D genes show 15 or 16 coding exons, with the notable exception of the mammalian MGAT4D having only 10 coding exons, whereas the coding sequence of MGAT4C, MGAT4F, MGAT4E, and MGAT4G genes is split into 2 to 3 exons. In addition to a cytoplasmic tail and a single pass transmembrane domain, all the GT54 open reading frames show a catalytic, a linker, and a lectin domain (CBM94) either spread on one or several exons but not the mammalian MGAT4D genes. During the evolution course, the mammalian MGAT4D gene ancestor lost its most 3′ exons encompassing the linker and lectin domain, which likely explains the absence of GnT-IV activity (i.e., β1,4-GlcNAc branching activity) of the human protein.^8^^,^^16^^,^^17^^,^^33^ The MGAT4 genes within each subgroup show conserved exon-intron boundaries, indicating their common origin. In the human genome, the MGAT4E and MGAT4F gene homologues are found on chromosome 1 but have lost their protein-coding capability during human evolution and were therefore named MGAT4EP and MGAT4FP. Interestingly, although MGAT4EP does not encode a protein, it is a functionally relevant unitary pseudogene (MGAT4EP) overexpressed as an ncRNA in breast cancer that upregulates the expression of oncogenic transcription factor FOXM1.^52^ Their protein-coding counterparts in the mouse genome are found on chromosome 1 (Figure 2). However, these two proteins likely have lost their catalytic function since the mouse MGAT4E and MGAT4F do not show the conserved DXD motif that is essential for UDP-GlcNAc and manganese coordination in the catalytic binding pocket. In addition, MGAT4E lost the exon encoding transmembrane domain. These findings denote an accumulation of disruptive mutations and a higher propensity of these genes to be lost along mammalian evolution.

#### Protein sequence analyses reveal two GT54 subgroups

As reported previously for several GTs of the GT-A fold,^53^ members of the GT54 are multi-modular proteins displaying additional structural domains beside their catalytic domain. The GT54 sequences contained a catalytic domain known as Glyco_transf_54 (PFAM domain PF04666), a linker of unknown function, and a C-terminal extension encompassing a lectin domain. The lectin domain present in mouse MGAT4A and MGAT4B was shown to be essential for their GnT-IV’s functions binding to their glycoprotein product (i.e., GlcNAc-terminated *N*-glycans on proteins).^8^^,^^44^^,^^46^ The mammalian MGAT4D proteins lack this lectin domain and are not able to catalyze the transfer of GlcNAc residues on *N*-glycosylated proteins (see functional analyses section below).

**Figure 3:**
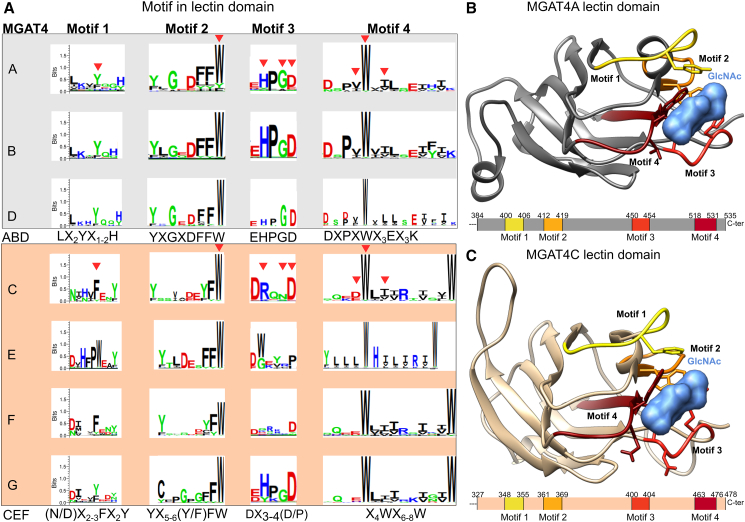
Conserved motifs in the MGAT4 lectin domain (A) Four conserved peptide motifs were identified within the lectin domain for each subfamily (MGAT4A to MGAT4G). Each subfamily’s motif is visualized by a sequence logo, which is a graphical representation of the information content (conservation and variability) of the motif in the MSA and which is generated using the Seq2Logo 2.0 program.^54^ The *y* axis describes the amount of information in bits. Subfamilies can be gathered into two subgroups: ABD (gray background) and CEFG (peach background). The amino acid residues of MGAT4 that interact with β-GlcNAc are indicated with a red triangle for MGAT4A and MGAT4C proteins. The consensus sequence for each subgroup is indicated below. Notably, MGAT4G shares the CEFG subgroup’s consensus sequence for motifs 1, 2, and 4 but aligns with the ABD subgroup’s consensus for motif 3. (B) Structural location of the four conserved ABD motifs, represented in the 3D model of the human MGAT4C lectin domain. Residues from the four conserved motifs are involved in GlcNAc binding, and GlcNAc-interacting residues are in stick representation. A schematic of the protein sequence below indicates the domain boundaries on the human sequence (UniProt ID: Q9UM21). (C) Structural location of the four conserved CEFG motifs, shown in the 3D model of the human MGAT4C lectin domain. Residues from the four motifs are involved in GlcNAc binding, and GlcNAc interacting residues are in stick representation. The schematic representation of the protein sequence below indicates the domain boundaries on the human sequence (UniProt ID: Q9UBM8).

**Figure 4:**
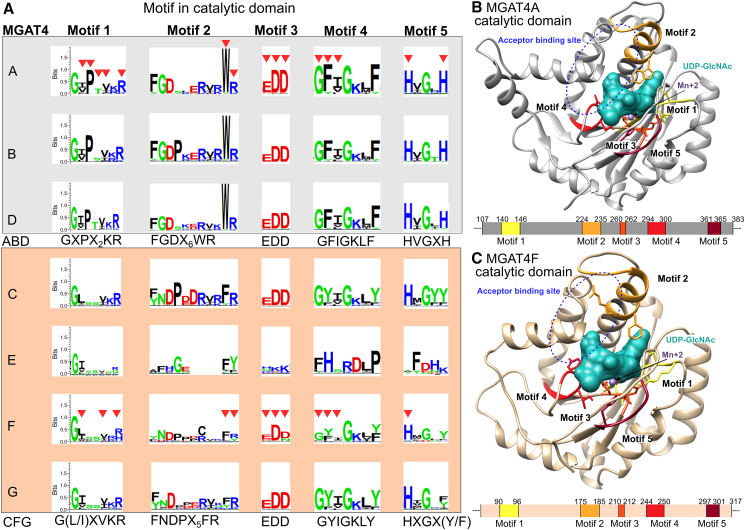
Conserved motifs in the MGAT4 catalytic domain (A) Five conserved motifs were identified within the catalytic domain for each subfamily (MGAT4A to MGAT4G). Each subfamily’s motif is visualized by a sequence logo, which is a graphical representation of the information content (conservation and variability) of the motif in the MSA and which is generated using the Seq2Logo 2.0 program.^54^ The *y* axis describes the amount of information in bits. Subfamilies can be divided into two subgroups: ABD (gray background) and CEFG (peach background). The residues interacting with UDP-GlcNAc are indicated with a red triangle for MGAT4A and MGAT4F. The consensus sequence for each group is displayed below. Notably, MGAT4E shows differences in motifs 2 to 5 compared with the other subfamilies. (B) Structural location of the five conserved ABD motifs, represented in the 3D model of the human MGAT4A catalytic domain. UDP-GlcNAc-interacting residues are in stick representation; residues from the five motifs are involved in UDP-GlcNAc binding. The putative localization of the substrate acceptor is encircled in blue. A schematic of the protein sequence below indicates the domain boundaries on the human sequence (UniProt ID: Q9UM21). (C) Structural location of the four CEFG motifs, shown in the 3D model of the chicken MGAT4F catalytic domain. UDP-GlcNAc-interacting residues are in stick representation; residues from the five motifs are also involved in UDP-GlcNAc binding. The schematic representation of the protein sequence indicates the domain boundaries on the chicken sequence (UniProt ID Q9DGD1).

The four human GT54 and representative protein sequences from the seven GT54 subfamilies described in the molecular phylogeny section were selected to generate multiple sequence alignments (MSA), allowing the identification of four well-conserved motifs in the lectin domain and five motifs in the catalytic domain that are GT54 signatures likely to be structurally and/or functionally significant (Figures 3 and 4, respectively). Sequence logos were created for each subfamily’s motif, providing a graphical representation of these evolutionary conserved motifs (Figure 3, Figure 4A and 4A). To investigate the potential structural and functional relevance of these conserved amino acid (aa) positions, we mapped them onto 3D models of representative human or chicken MGAT4 proteins from each subgroup.

In the lectin domain, the first motif LX_2_YX_1-2_H in the ABD subgroup contains a highly conserved tyrosine residue (Y), which is absent in the corresponding motif (N/D)X_2-3_FX_2_Y of the CEFG subgroup (Figure 3A). The second motif, YXGXDFFW in the ABD and (YX_5-6_(Y/F)FW) in the CEFG, is well conserved, notably preserving a highly conserved tryptophan residue (W) in the final position (Figure 3A). The third motif EHPGD, conserved in all the subfamilies of the ABD, diverges in the CEFG to DX3-4(D/P) except for the last aspartate residue (D), which is not conserved in the MGAT4E proteins (Figure 3A). The fourth motif DXPXWX3EX3K well conserved in the ABD shows divergence in the CEFG, retaining a W residue in the middle of the motif (X4WX6-8W) (Figure 3A). These four conserved motifs were mapped onto the 3D structural models of lectin domain with β-GlcNAc of either human MGAT4A (UniProt ID: Q9UM21) (Figure 3B) or human MGAT4C (UniProt ID: Q9UBM8) (Figure 3C). They were predicted to participate in GlcNAc binding. The four highly conserved aa positions in each motif correspond to GlcNAc-interacting residues represented in stick form in Figures 3B and 3C. Moreover, these aa positions correspond to the well-conserved aa Y394, W410, D445, and W513 in mouse MGAT4A (UniProt ID Q812G0-2)^44^ and in the bacterial GlcNAc-binding lectin NagH.^55^

Five evolutionary conserved peptide motifs were identified in the catalytic domain of MGAT4 proteins (Figure 4). The first conserved motif GXPX2KR in the ABD subgroup contains a conserved aliphatic residue isoleucine/valine/leucine (I/V/L) immediately preceding the K/R residues and has a corresponding form G(L/I)XVKR in the CEFG subgroup (Figure 4A). In 3D models of the catalytic domains, this first motif is positioned near the sugar donor UDP-GlcNAc, within the catalytic site in both human MGAT4A (UniProt ID: Q9UM21) and chicken MGAT4F (UniProt ID: Q9DGD1) (Figures 4B and 4C). The second conserved motif FGDX6WR in the ABD or FNDPX5FR in the CEFG contains a conserved aromatic residue (either W or F) (Figure 4A) that may interact with UDP-GlcNAc (Figures 4B and 4C). This second motif also includes a conserved arginine (R) with potential structural implication in the acceptor binding site (Figure 4). The third conserved motif identified in the GT54 proteins corresponds to the well-described DxD motif known to be implicated in the coordination of a metal ion (i.e., Mn^2+^) alongside the sugar donor of the majority of GT-A fold GTs (Figures 4B and 4C). The fourth motif GFIGKLF in the ABD and GYIGKLY in the CEFG is highly conserved within each subgroup except for MGAT4E. It is positioned in the substrates binding site where its conserved residues likely interact with both donor and acceptor substrates (Figures 4B and 4C). The fifth motif, HVGXH in the ABD and HXGX(Y/F) in the CEFG, shows a histidine residue (H) that is highly conserved in all the GT54 proteins except MGAT4E (Figures 4B and 4C). Conserved motifs of the catalytic domain likely have essential structural and functional importance, as they are located nearby the DxD motif in the active site, forming cavities where the carbohydrate substrates could bind. They are highly conserved in the first ABD subgroup and are more rapidly evolving in the CEFG. In particular, the mammalian MGAT4E and MGAT4F proteins present highly modified motifs, suggesting the loss of catalytic function of these mammalian GT54. As an example, mouse MGAT4F shows no conserved motifs and only 35% sequence identity with the chicken MGAT4F protein.

Collectively, these sequence-based analyses provided indications on the functional importance of each domain (lectin and catalytic), and predicted evolutionary conserved sequence motifs will shed light into the structural and functional organization of these glycosyltransferases. These in-depth analyses enabled us to gather subfamilies into two subgroups, ABD and CEFG, and they indicated marked differences in both the lectin and catalytic domains of each GT54 subgroup (Figure S1) that could be critical for their enzymatic specificity. Although the underlying molecular bases remain to be established, these sequence-based analyses unveil a common ancestry of the identified sequences and their functional divergence in early Metazoa.

#### Existence of additional vertebrate MGAT4 subfamilies lost in mammals

**Figure 5:**
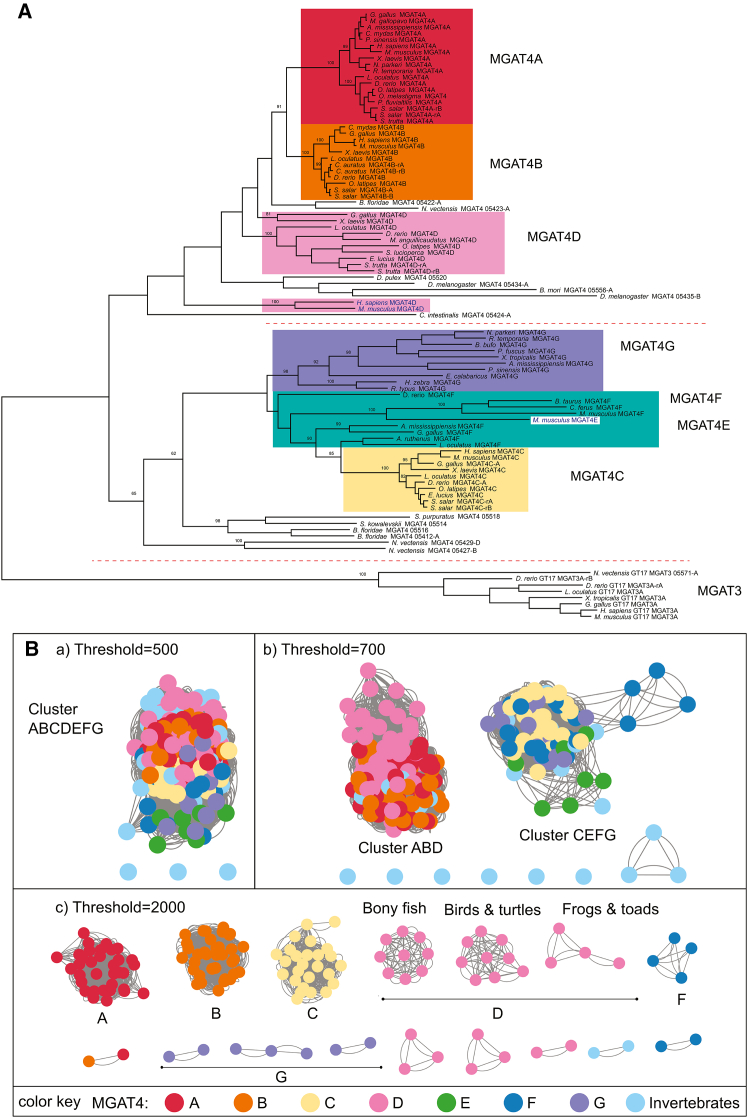
ML phylogenetic tree of 92 GT54-related sequences from Metazoa (A) Maximum likelihood (ML) tree was constructed using MEGA 11.0^57^ based on the JTT matrix-based model.^58^ The tree with the highest log likelihood (−55845.83) is shown. Ninety-two GT54-related sequences from Metazoa *Bos taurus* (*B. taurus*), *Homo sapiens* (*H. sapiens*), *Mus musculus* (*M. musculus*), *Xenopus laevis* (*X. laevis*), *Gallus gallus* (*G. gallus*), *Meleagris gallopavo* (*M. gallopavo*), *Alligator mississippiensis* (*A. mississippiensis*), *Chelonia mydas* (*C. mydas*), *Pelodiscus sinensis* (*P. sinensis*), *Nanorana parkeri* (*N. parkeri*), *Rana temporaria* (*R. temporaria*), *Lepisosteus oculatus* (*L. oculatus), Danio rerio* (*D. rerio*), *Oryzias latipes* (*O. latipes*), *Oryzias melastigma* (*O. melastigma*), *Perca fluviatilis* (*P. fluviatilis*), *Salmo salar* (*S. salar*), *Salmo trutta* (*S. trutta*), *Carassius auratus* (*C. auratus*), *Branchisotoma floridae* (*B. floridae*), *Nematostella vectensis* (*N. vectensis*), *Misgurnus anguillicaudatus* (*M*. *anguillicaudatus*), *Sander lucioperca* (*S. lucioperca*), *Eso*x *lucius* (*E. lucius*), *Daphnia pulex* (*D. pulex*), *Drosophila melanogaster* (*D. melanogaster*), *Bombyx mori (B. mori*), *Ciona intestinalis* (*C. intestinalis*), *Bufo bufo* (*B. bufo*), *Pelobates fuscus* (*P. fuscus*), *Xenopus tropicalis* (*X. tropicalis*), *Erpetoichthys calabaricus* (*E. calabaricus*), *Heterodontus zebra* (*H. zebra*), *Rhincodon typus* (*R. typus*), *Camelus ferus* (*C. ferus*), *Acipenser ruthenus* (*A. ruthenus*), *Strongylocentrotus purpuratus* (*S. purpuratus*), *Saccoglossus kowalevskii* (*S. kowalevskii*), and eight GT17 MGAT3 sequences used as an outgroup were selected for multiple sequence alignments performed with the MUSCLE algorithm^59^ (Data S1, Table S1). There was a total of 800 positions in the final dataset. The topology of the ML tree indicates two deeply rooted branches corresponding to the subgroups ABD and CEFG. The seven GT54 orthologue groups are split into two subgroups: the ABD subgroup comprised of MGAT4A (clade 1), MGAT4B (clade 2), and MGAT4D (clade 3) on one hand and the CEFG subgroup comprised of MGAT4C (clade 4), MGAT4E (clade 5), MGAT4F (clade 6), and MGAT4G (clade 7) on the other hand. The major vertebrate branches and bootstrap support values of more than 60% are indicated. The ML tree with statistical support (bootstraps and branch length) is shown in Figure S3. (B) Clustering of metazoan MGAT4 sequences from the GT54 family identifies two clusters. Sequence Similarity Network (SSN) with nodes representing individual MGAT4 proteins and edges representing pairwise alignment bit scores. (A) At a threshold of 500, nearly all GT54 proteins are connected by edges, forming a single large cluster, indicating their overall similarity. (B) At a threshold of 700, two main clusters are formed. One cluster includes the MGAT4A, MGAT4B, and MGAT4D sequences, while the other includes the MGAT4C, MGAT4E, MGAT4F, and MGAT4G sequences. Both clusters include sequences from invertebrates. (C) At a threshold of 2,000, sequences further split into clusters corresponding to subfamilies (e.g., MGAT4A, MGAT4B, and MGAT4C). Some subfamilies, which seem to be evolving faster, are divided into several smaller clusters (e.g., MGAT4D and MGAT4G). Singletons are omitted.

To gain insights into the broad and diverse GT54 repertoire, we assessed the homology of vertebrate and invertebrate MGAT4-related protein sequences by MSA and phylogeny analysis. The first analysis was conducted using IQ-TREE,^56^ with a large dataset of 231 MGAT4-related sequences spanning the diversity of Metazoa and including 41 invertebrate sequences (Figure S2). To achieve correct rooting of our GT54 phylogenetic trees, a set of more distantly related sequences (MGAT3)^42^ was chosen, and for the sake of clarity, maximum likelihood (ML) trees were constructed using 92 selected sequences both from vertebrate and invertebrate species. The resulting tree in Figure 5A clearly organizes the GT54-related sequences in two deeply rooted branches gathering MGAT4A (clade 1), MGAT4B (clade 2), and MGAT4D (clade 3) on one hand and MGAT4C (clade 4), MGAT4E (clade 5), MGAT4F (clade 6), and MGAT4G (clade 7) on the other. The split between the two subgroups predates the emergence of Cnidaria (∼824 million years ago, MYA) since the starlet sea anemone *N. vectensis* or the hydra *Hydra vulgaris* already possess several sequences representative of each subgroup (Figures 5A and S2), and no MGAT4-related sequence could be identified in nonmetazoan species. The seven GT54 orthologue groups emerge later on, at the dawn of vertebrates. We also assessed the robustness of this phylogenetic tree, which is displayed with statistical support and branch length in Figure S3.^56^

The first cluster gathers vertebrate MGAT4A, MGAT4B, and MGAT4D sequences. It is rooted by invertebrate sequences from the tunicate *Ciona intestinalis*, the cephalochordate *Branchiostoma floridae*, the cnidarians *N. vectensis*, *E. diaphana*, and *P. damicornis*, the poriferans *Corticium candelabrum* and *Oscarella lobularis*, the placozoan *Trichoplax adhaerens*, the nematodes *Trichuris suis* and *Brugia malayi*,^60^ and the arthropods *Drosophila melanogaster*, *Apis mellifera*, *Daphnia pulex*, and *B. mori*, which are orthologous to the common ancestor sequences of vertebrate clusters MGAT4A, MGAT4B, and MGAT4D (Figures 5A and S2). The topology of this branch also uncovered that the MGAT4D genes emerged first at the stem of vertebrates likely after the first round of whole-genome duplication WGD-R1 (∼550 MYA) and underwent faster changes, whereas the other two subfamilies, MGAT4A and MGAT4B, emerged more recently after the second round of WGD-R2 (∼500 MYA) and were subject to evolutionary constraints during vertebrate evolution. While the vertebrate MGAT4A and MGAT4B show short branches and are representative of phylogenetic relationships between species, the vertebrate MGAT4D shows a scattered pattern and longer branches (Figures 5A, S2, and S3). Orthologues of the MGAT4A, MGAT4B, and MGAT4D could be identified in most investigated ray-finned fish species. Salmonid fishes like *Salmo salar* and *Oncorhynchus mykiss* showed two paralogous MGAT4A, MGAT4B, and MGAT4D sequences, whereas the other fish like the zebrafish *Danio rerio* showed only one orthologue. The position of these duplicated genes in the phylogenetic trees (Figures 5A and S2) indicated that they likely resulted from the more recent Salmonidae-specific genome duplication event WGD-R4 (SGD or Ss4R), which took place ∼80 MYA^61^ and have been maintained in salmoniformes genome. Finally, the fish MGAT4D sequences, previously known as MGAT4B-like sequences, remained relatively close to MGAT4B, whereas mammalian MGAT4D showed long branches, do not cluster with the other MGAT4D sequences, and were artifactually placed outside the rest of the clade. This observation is likely to be correlated to the loss of the lectin domain of mammalian MGAT4D and further suggests the loss of activity of these proteins during mammalian evolution.

The second cluster gathers vertebrate MGAT4C, MGAT4E, MGAT4F, and MGAT4G sequences, and this subgroup is also rooted by invertebrate sequences from *N. vectensis*, *E*. *diaphana*, *B. floridae*, *O. lobularis*, *C. candelabrum*, and *T. adhaerens* and the Echinodermata Echinoidea *Strongylocentrotus purpuratus* and *Lytechinus variegatus variegatus* (sea urchin) and the Echinodermata Asteroidea *Acanthaster planci* and *Asterias rubens* and the hemichordate *Saccoglossus kowalevski* (Figures 5A and S2). Analysis of this cluster reveals an explosive acceleration of evolution of these sequences presumably associated with pseudogenization of genes and/or functional shift of enzyme and protein loss. The MGAT4G clade likely emerged first, as a result of WGD-R1, and underwent faster changes as evidenced by the long branches that led to inactivation and/or independent gene loss in many lineages (Figure S3). The MGAT4G orthologue is found in neither mammalian, avian, nor ray-finned fish genomes but is still present in sharks, some amphibians, and sauropsid (reptiles and birds) genomes (Figures 5A and S2). The sister branch of MGAT4G encompasses MGAT4C, MGAT4E, and MGAT4F clades. The MGAT4C clade likely emerged after the WGD-R2. Short branches and presence of this orthologue in all the studied vertebrate species indicate selective pressure against mutations and pseudogenization in this clade. The presence of MGAT4C duplicates in most ray-finned fish genomes, but not in the non-teleost fish *Lepisosteus oculatus*, strongly suggests that a paralog arose from the third WGD event (WGD-R3 or TGD) in teleosts that took place 320–350 MYA.^61^ The MGAT4F clade is dispersed in all the vertebrates with the exception of amphibians, and the orthologue is found in a restricted number of animal species. Of particular interest is the presence of a MGAT4F orthologue in chicken corresponding to the previously described chicken MGAT4C-like (MGAT6)^19^^,^^34^^,^^35^^,^^36^ that is distinct from the chicken MGAT4C gene (Figures 5A and S2). MGAT4E and MGAT4F subfamily members do not cluster as distinct clades, and the mammalian MGAT4E sequences are found inserted into the mammalian MGAT4F clade in the phylogenetic trees. Since MGAT4E genes are tandemly arrayed with MGAT4F on the same mammalian chromosome and are not found in other vertebrate branches, they likely originate from a tandem duplication event of MGAT4F that occurred at the base of mammals.

To provide a more detailed view of sequence similarity relationships across this diverse GT54 multidomain protein family in a larger set of full-length sequences, we used the complementary Sequence Similarity Network (SSN)^62^ approach, which enabled us to visualize clusters and subgroups based on sequence similarity. We analyzed 218 sequences using three different bit-score thresholds: 500, 700, and 2,000 (Figure 5B). Nodes were colored according to MGAT4 subfamily (A–G) or invertebrate sequences. At a threshold of 500, almost all sequences clustered together (Figure 5Ba). When the threshold was raised to 700, two major clusters emerged, one corresponding to ABD and the other to CEFG subgroup, supporting the existence of two main evolutionary subgroups or families within GT54 (Figures 5A and 5B). Some invertebrate sequences appeared as singletons, meaning they did not meet the similarity criteria to form connections with other sequences. At the highest threshold of 2,000, distinct clusters emerged for subfamilies MGAT4A, MGAT4B, and MGAT4C, while subfamily MGAT4D split into six clusters, reflecting a lower degree of similarity among its sequences (Figure 5B and 5C). Subfamilies MGAT4G and MGAT4F were further divided into two and three clusters, respectively. All members of subfamily MGAT4E appeared as singletons, indicating a particularly low level of similarity. Overall, these SSN results provide strong support for the existence of two primary GT54 subgroups composed of ABD and CEFG subfamilies, with notable lower sequence similarity within the CEFG cluster.

#### Comparative genomics clarifies evolutionary relationships and functional divergence of GT54 families

The WGD events that occurred before the emergence of jawless fish-like lampreys provided an important source of duplicate genes,^63^^,^^64^ with a major impact on species evolution. These genetic events resulted in large multigene families of paralogues, which sometimes experienced gene losses, and subsequently, duplicated genes eventually diverged in various protein functions.^65^ Since phylogenetic trees sometimes suggest an orthology for a particular pair of genes, which will later turn out to be paralogous, it is important to infer gene homology and identify ohnologue pairs using paralogy and synteny analyses and conserved genomic context. To clarify the evolutionary relationships of the vertebrate GT54-related clades and assess common ancestry of non-clustering clades like vertebrate MGAT4D or MGAT4F, we search for the existence of paralogons (i.e., chromosome segments affected by WGD in vertebrates). Using the Synteny Database site,^66^ eight paralogon pairs were found in the human genome on chromosomes 1, 2, 4, 5, 11, and 12 bearing the six human gene loci MGAT4EP and MGAT4FP, MGAT4A, MGAT4D, MGAT4B, MGAT4C, and the lost MGAT4G, respectively, and INPP4A/INPP4B, TBC1D8/TBC1D9B, RNF130/RNF150, RFX8/RFX4, LSM6/SNRPF, ADIPOR2/ADIPOR1, RASSF9/RASS10, TEAD4/TEAD1, SYT10/SYT2, IPO8/IPO7, and ALX1/ALX3 (Figure S4). The existence of human paralogons between members of the two GT54 subgroups ABD and CEFG is indicative of an ancient tandem duplication followed by genomic rearrangements as suggested by our synteny analysis around the invertebrate *C. intestinalis* MGAT4 loci (Figure S5).

We also assessed synteny (i.e., blocks of orthologous genes) between vertebrate GT54-related and adjacent genes to GT54 gene loci in eight vertebrate taxa. A well-conserved synteny could be established for the vertebrate MGAT4A, MGAT4B, MGAT4D, and MGAT4C gene loci corroborating the phylogenetic trees topology. As the MGAT4G gene was absent in mammals, birds and fish, we considered the neighboring genes TEAD1, MICAL2, and RASSF10 of the *mgat4g* gene locus present on *X. tropicalis* chromosome 4 to retrieve the synteny on human (Hsap11), mouse (Mmus7), chicken (Ggal5), spotted gar (LocuLG27), and salmon (Ssal10 and Ssal16) chromosomes (Figure S6). Similarly, for the *mgat4f* gene absent in amphibians and fish, the TMEM183A, PPFIA4, and MYOG in the vicinity of the human MGAT4FP locus on chromosome 1 (Hsap1) were used to retrieve the synteny on the frog (Xtro2), medaka (Olat5), and salmon (Ssal22 and Ssal12) chromosomes.

To certify our phylogeny reconstruction and paralogy relationships data, we used ancestral genome reconstruction of the vertebrate and chordate ancestor according to the N-model^67^ and the P-model.^68^ 2R-duplicated genes are found on one of the 10 vertebrate ancestral chromosomes (VAC) in the pre-2R genome and designated A–J in the N-model.^67^ Similarly, they are found on one of the 17 hypothetical ancestral chordate linkage groups (CLG) in the pre-2R genome numbered A to Q in the P-model^68^^,^^69^ and on one of the 13 teleost ancestral proto-chromosomes named A to M in the pre-3R genome reconstructed from the extant fish genomes.^70^ After the two WGD-2R events, they are found on four linkage groups with shared synteny. Conserved synteny was established for MGAT4A, MGAT4B, and MGAT4D, and surrounding gene loci and the blocks associated to these genes corresponded to the CLG proto-chromosome F in the P-model and to the ancestral proto-vertebrate chromosome Conserved Linkage Group C (CLG-C) in the N-model located on gnathostome ancestor (GNA) proto-chromosomes C1 and C2 (it could not be assigned for MGAT4A) (Figure S7). Conserved synteny was established for MGAT4C, MGAT4F, and MGAT4G, and surrounding gene loci and the blocks associated to these genes corresponded to the CLG proto-chromosome E/O in the P-model and to the ancestral proto-vertebrate chromosome CLG-D in the N-model located on GNA proto-chromosomes D0, D2, and D1 (Figure S7). A copy of the MGAT4A/B/D orthologue is localized on the amphioxus chromosome Bfl7 (Figure S8), which is a descendant of the proto-vertebrate chromosome CLG-C, the bilaterian ancestral linkage group 1 (B.ALG1), and the metazoan ALG6.^71^^,^^72^ Conserved synteny was also established for MGAT4C, MGAT4F, and MGAT4G, and surrounding gene loci and the blocks associated to these genes corresponded to GNA proto-chromosomes D0, D2, and D1 and to the CLG proto-chromosome E/O (Figure S7). The ancestral MGAT4C/E/F/G gene was located on CLG-D (Figure S6), and the *B. floridae* chromosome Bfl4 issued from the CLG-D bears three copies of the MGAT4C/E/F/G orthologue (Figure S7). This genome reconstruction approach definitively confirmed the common origin of the MGAT4A, MGAT4B, and MGAT4D clades on one hand, and the MGAT4C, MGAT4F, and MGAT4G clades on the other, supporting the proposed evolutionary scenario in which the GT54 family originated from two ancestral MGAT4 genes already present at the dawn of Metazoa.

#### Functional characterization of two distinct GnT-IV specificities

**Figure 6:**
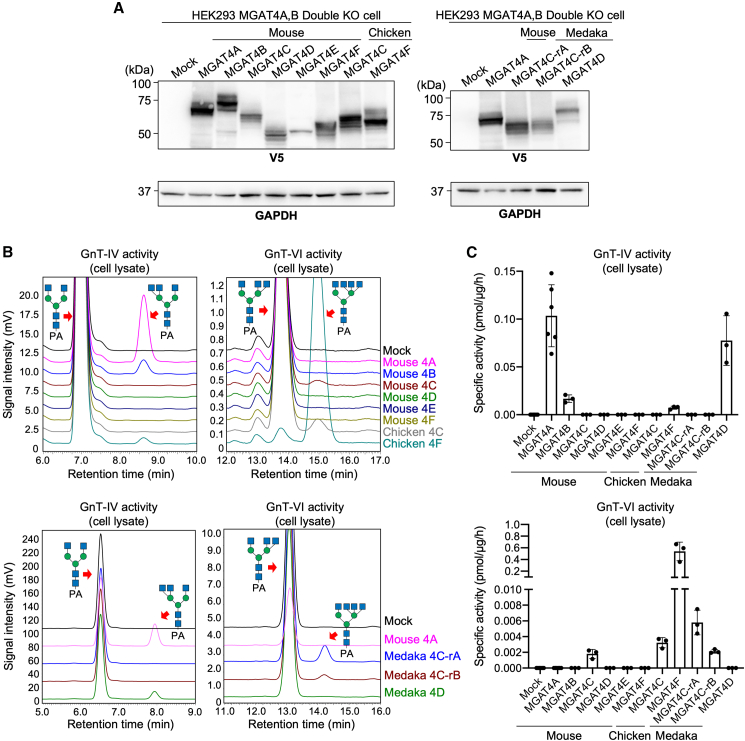
Enzymatic activity of mouse MGAT4A-F, chicken MGAT4C and MGAT4F, and medaka MGAT4C-rA, MGAT4C-rB, and MGAT4D (A) Proteins from MGAT4A/MGAT4B DKO HEK293 cells transfected with an empty vector (mock) or a plasmid for expression of mouse MGAT4A-F, chicken MGAT4C or MGAT4F, or medaka MGAT4C-rA, MGAT4C-rB, or MGAT4D were subjected to SDS-PAGE and blotted with anti-V5 antibody (upper) or anti-GAPDH antibody (lower). (B) Overlay of HPLC chromatograms for acceptor substrates and branched *N*-glycan products in MGAT4 reactions. Lysates of DKO cells transfected with each plasmid were reacted with fluorescence (PA, 2-aminopyridine)-labeled bi-antennary *N*-glycan (left) or tri-antennary *N*-glycan with β1,6-GlcNAc on α1,6-Man (MGAT5 product) (right). The reaction mixtures were analyzed by reversed phase HPLC to separate the substrates and the products. The elution positions of the substrates and the expected products are indicated by red arrows.^73^ (C) Specific activity of the lysates expressing each enzyme is shown (*n* = 3, mean ± SD).

Molecular cloning of the mouse MGAT4A, MGAT4B, MGAT4D, MGAT4C, MGAT4E, and MGAT4F, the chicken MGAT4C and MGAT4F, and the medaka MGAT4C-rA, MGAT4C-rB, and MGAT4D sequences was carried out, and recombinant proteins were produced in MGAT4A and MGAT4B double-knockout (DKO) HEK293 cells (Figure 6A). To measure their enzymatic activity, lysates of transfected DKO cells were incubated with fluorescent (PA, 2-aminopyridine)-bi-antennary *N*-glycan and tri-antennary *N*-glycan with β1,6-GlcNAc on α1,6-Man (MGAT5 product) and analyzed using reversed phase HPLC. We checked that the mouse enzymes had a GnT-IV-like activity on biantennary *N*-glycan substrate, thereby forming tri-antennary *N*-glycans (Figures 6B and 6C, upper). Contrasting with the mammalian MGAT4A, MGAT4B activity, the chicken MGAT4F recombinant protein preferred the tri-antennary *N*-glycan with β1,6-GlcNAc on α1,6-Man (i.e., MGAT5 product) as an acceptor substrate rather than the bi-antennary *N*-glycan (Figures 6B, 6C, and S9) and showed a GnT-VI-like activity as described previously.^19^ To our surprise, although the activity of human MGAT4C has not been detected previously,^19^ we detected a small amount of product formed by mouse and chicken MGAT4C (Figures 6B, 6C, and S9), suggesting that these MGAT4C proteins also have weak enzyme activity. However, mouse MGAT4F and MGAT4E showed no GnT-IV- nor GnT-VI-like activity on these two acceptor substrates, which may be attributed to accumulation of changes in these two MGAT4 sequences, in particular in the evolutionary conserved motifs described above.

We also assayed the transfer activity of medaka MGAT4C-rA, MGAT4C-rB, and MGAT4D, since MGAT4F is not present in this species. Unlike mammalian MGAT4D, medaka MGAT4D has a lectin domain, and we observed the reaction product of medaka MGAT4D with the same retention time and mass as those of mouse MGAT4A (Figures 6B, lower left panel, andS9). These data strongly suggested that fish MGAT4D is an active enzyme with the same specificity as MGAT4A and MGAT4B. Furthermore, we found that both medaka MGAT4Cs also showed weak activity with similar specificity to mouse and chicken MGAT4C (Figures 6C, lower panel, and S9).

**Figure 7:**
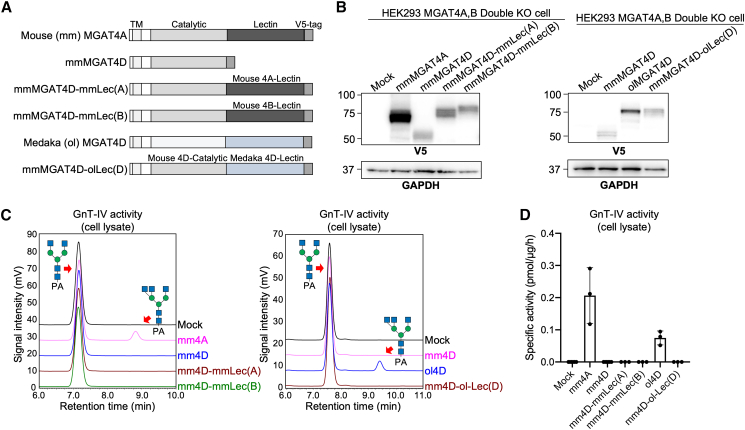
Enzymatic activity of chimeric mouse MGAT4D (A) Plasmid constructs used in this study. (B) Proteins from MGAT4A/MGAT4B DKO HEK293 cells transfected with an empty vector (mock) or a plasmid for expression of mouse (mm) MGAT4A, mmMGAT4D, mmMGAT4D-mmLec(A), mmMGAT4D-mmLec(B), medaka (ol) MGAT4D, or mmMGAT4D-olLec(D) were subjected to SDS-PAGE and blotted with anti-V5 antibody (upper) or anti-GAPDH antibody (lower). (C) Overlay of HPLC chromatograms for acceptor substrates and branched *N*-glycan products in MGAT4 reactions. Lysates of DKO cells transfected with each plasmid were reacted with fluorescence (PA, 2-aminopyridine)-labeled bi-antennary *N*-glycan (left) or tri-antennary *N*-glycan with β1,6-GlcNAc on α1,6-Man (MGAT5 product) (right). The reaction mixtures were analyzed by reversed phase HPLC to separate the substrates and the products. The elution position of the substrates and the expected products are indicated by red arrows. (D) Specific activity of the lysates expressing each enzyme is shown (*n* = 3, mean ± SD).

Our enzyme assay showed that medaka MGAT4D bearing lectin domain is active (Figure 6B), which raised a possibility that the loss of activity of mammalian MGAT4D is caused by the loss of lectin domain. To test this possibility, we generated constructs for expressing chimeric mouse MGAT4D proteins having the lectin domain of mouse MGAT4A or mouse MGAT4B or medaka MGAT4D (Figure 7A). These chimeric enzymes were expressed in DKO cells (Figure 7B), and their *in vitro* activity was assessed using cell lysates. However, none of these chimeric proteins exhibited enzymatic activity (Figures 7C and 7D). This suggests that the loss of activity of mammalian MGAT4D is not solely caused by the loss of lectin domain.

Collectively, these experimental data indicated the existence of two functional subgroups of metazoan GT54 enzymes: the evolutionary conserved α1,3-mannosyl-glycoprotein β1,4-*N*-acetylglucosaminyltransferases subgroup encompassing the vertebrate MGAT4A, MGAT4B, and MGAT4D and invertebrate MGAT4A/B/D proteins and the α1,6-mannosyl-glycoprotein β1,4-*N*-acetylglucosaminyltransferases subgroup gathering the fast-evolving vertebrate MGAT4C, MGAT4E, MGAT4F, and MGAT4G and the invertebrate MGAT4C/E/F/G proteins. In terms of functional organization of this Golgi machinery of glycosylation, our data further suggest that GnT-IV-like enzymes of the ABD subgroup chronologically act after β1,2-*N*-acetylglucosaminyltransferase MGAT1 and sometimes after MGAT2, whereas those belonging to the CEFG subgroup act after α1,6-mannosyl-glycoprotein β1,6-*N*-acetylglucosaminyltransferase MGAT5. In this connection, several studies have provided evidence for the formation in the Golgi membranes of macromolecular assemblies involving GT54 enzymes of the ABD subgroup, MGAT1, MGAT2, MGAT3 (but not MGAT5), the alpha-mannosidase IIX (MAN2A2), and sugar nucleotide transporters (NST), including the UDP-GlcNAc transporter SLC35A3.^16^^,^^74^^,^^75^^,^^76^ These assemblies modulate MGAT enzymatic activities either facilitating or inhibiting efficient *N*-glycan branching. Although it requires further exploration, our data suggest that the vertebrate GT54 enzymes of the CEFG subgroup would rather interact with MGAT5 and SLC35A3 and subsequent actors of the *N*-glycan biosynthesis pathway like the UDP-Gal transporter SLC35A2 and galactosyltransferases, thereby regulating this pathway.

### Conclusion

**Figure 8:**
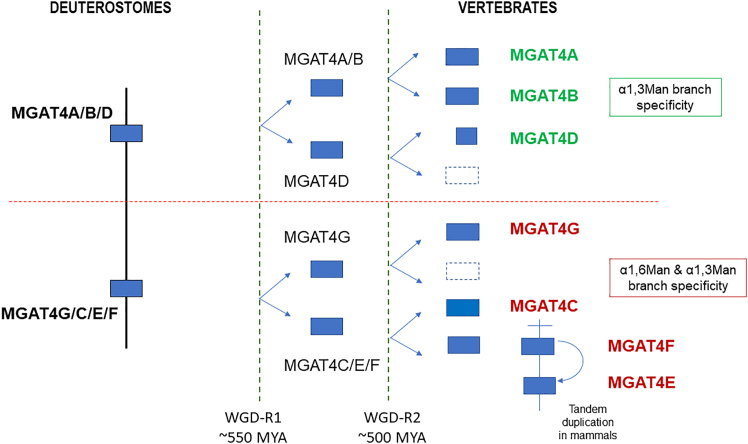
Scenario of the evolutionary relationships of metazoan MGAT4 genes This schematic depicts a model for the evolution of the seven orthologue groups of vertebrate MGAT4 genes (MGAT4A, MGAT4B, MGAT4C, MGAT4D, MGAT4E, MGAT4F, and MGAT4G). The proposed scenario is based on evidence from phylogenetic analysis, and it takes into account the two rounds of whole-genome duplication WGD-2R (WGD-R1 550 MYA and WGD-R2 500 MYA) that took place early in the vertebrate lineage. It suggests the existence of two ancestral genes localized on the same ancestral proto-chromosome (prechordate) and two subgroups ABD and CEFG in early metazoans (e.g., Porifera and Cnidaria) and the emergence of seven orthologue groups in vertebrates. Among these seven clades, MGAT4E likely results from a duplication and translocation event early in the ancestor of mammals. Across vertebrate evolution, some clades were maintained in all the branches like MGAT4A and MGAT4B, while some other disappeared like MGAT4G and MGAT4F in mammals. The dark blue boxes indicate the presence of the gene, whereas the white boxes represent gene loss in extant species.

The set of MGAT4 involved in the synthesis of branched *N*-glycans have remained poorly characterized to date. In this work, we used an integrated strategy combining genomics and functional approaches to study GT54 CAZymes, leading to major basic discoveries revolving around the branched *N*-glycan pathway and evolution of Metazoa. Although the origin of GT54 at the dawn of multicellular organisms remains uncertain, we uncovered the existence of two subgroups ABD and CEFG of GT54 with distinct enzymatic specificities in early Metazoa. Considering the role played by branched *N*-glycans on biological functions of cell adhesion molecules, it is tempting to speculate that GT54 genes participated in the integrin-mediated adhesion and signaling machinery, triggering the unicellular-to-multicellular transition that gave rise to metazoans as proposed before.^77^ These two GT54 subgroups or families further diversified after the emergence of vertebrates and the WGD rounds in three and four clades, respectively. The vertebrate ABD comprised of MGAT4A, MGAT4B, and MGAT4D is slowly evolving, supporting the idea of essential biological functions of these MGAT4 and tri-antennary *N*-glycans in vertebrates. On the other hand, the vertebrate CEFG encompassing MGAT4C, MGAT4E, MGAT4F, and MGAT4 G is rapidly evolving in the vertebrate lineages; it has undergone several independent genetic events of gene duplication and loss over the course of vertebrate evolution. These data point to a loss of function of GT54 with GnT-VI-like activity during mammalian evolution, suggesting that this *N*-glycan branching machinery could have been shaped by host-pathogen interaction in the course of mammalian evolution.^78^ Figure 8 illustrates a plausible scenario of their evolutionary relationships. Our analysis also helped to fully resolve the *N*-glycan biosynthetic pathway, elucidating the branching capabilities and substrate preference of several GT54-related sequences and enlightening our understanding of the functional organization of this glycosylation machinery in Golgi membranes. Altogether, these findings hold the potential to enhance future biotechnological applications, including the design and engineering of MGAT4 for specific glycosylation reactions.

### Limitations of the study

Having access to early Metazoa genomic data for phylogenomic analyses is a limitation of the phylogenomic study. Producing sufficient amount of active enzymes and having the right substrates are limitations in the biochemical study of MGAT4.

## Resource availability

### Lead contact

Further information and requests for resources and reagents should be directed to and will be fulfilled by the lead contact, Dr. Anne Harduin-Lepers (anne.harduin-lepers@univ-lille.fr).

### Materials availability

Reagents generated in this study are available from the lead contact with a completed Materials Transfer Agreement.

### Data and code availability

• This paper analyzes existing, publicly available data that are listed in the key resources table. The structural data used in this study from the Protein DataBank are listed in the key resources table.
• No original code was reported in this paper.
• Any additional information required to reanalyze the data reported in this paper is available from the lead contact upon request.

## Acknowledgments

The authors acknowledge the support of the University of Lille, the Center National de le Recherche Scientifique (CNRS), and the Japan Society for the Promotion of Science (JSPS).

This work was supported by a FOREST grant (JPMJFR215Z to Y.K. and JPMJFR2255 to H.Y.) from the 10.13039/501100002241Japan Science and Technology Agency, Grants-in-Aid for Scientific Research (B) (24K02222 to Y.K. and 25K02410 to H.Y.), a Core-to-core program (JPJSCCA202000007) from the 10.13039/501100001691Japan Society for the Promotion of Science (JSPS), and an AMED-CREST grant (JP23gm1410011 to Y.K.) from the 10.13039/100009619Japan Agency for Medical Research and Development (AMED). RET’s work was supported by the ANR-21-CE44–0032 (project PsaMar to A.H.-L.) from the 10.13039/501100001665Agence Nationale de la Recherche (ANR).

## Author contributions

Y.K. and A.H.-L. designed the study. A.M., R.E.T., M.N., H.Y., Y.K., and A.H.-L. collected and/or analyzed data and wrote the manuscript. Y.K. and A.H.-L. reviewed and edited the manuscript. All authors contributed to the article and approved the submitted version.

## Declaration of interests

The authors declare no conflict of interest.

## STAR★Methods

### Key resources table

**Table:** 

REAGENT or RESOURCE	SOURCE	IDENTIFIER
**Antibodies**		

Mouse anti-V5	Proteintech	v5ab; RRID: AB_2868500
Mouse anti-GAPDH (6C5)	Millipore	MAB374; RRID: AB_2107445

**Bacterial and virus strains**		

XL-10 Gold Ultracompetent cells	Agilent Technologies	200315
DH5-alpha competent cells	TAKARA	9057

**Chemicals, peptides, and recombinant proteins**		

Protease inhibitor cocktail set V (EDTA-free)	Fujifilm	168–26033
Lipofectamine 3000	ThermoFisher Scientific	L3000
NEBuilder HiFi DNA Assembly Master Mix	New England Biolabs	E2621
GnGnbi-PA	Takamatsu et al.^73^	N/A

**Critical commercial assays**		

Pierce BCA Protein Assay Kit	ThermoFisher Scientific	23227

**Experimental models: Cell lines**		

HEK293-GnT-IVa/GnT-IVb-DKO	Nagae et al.^44^	N/A
COS7	RIKEN Cell bank	RCB0539

**Oligonucleotides**		

Oligonucleotides for constructing the plasmids, see Table S2	This study	N/A

**Recombinant DNA**		

pcDNA6 myc-His A/mouse MGAT4A	Nagae et al.^44^	N/A
pcDNA-IH/mouse MGAT4A (Ser60-C-term)	Nagae et al.^44^	N/A
pcDNA6 myc-His A/mouse MGAT4B	Osada et al.^31^	N/A
pcDNA-IH/human MGAT5 (Thr121-C-term)	Vibhute et al.^79^	N/A
pBlueScript/chicken MGAT4F	Sakamoto et al.^19^	N/A
pcDNA6 myc-His A/mouse MGAT4C	This study	N/A
pcDNA6 myc-His A/mouse MGAT4D	This study	N/A
pEF1 V5-His A/mouse MGAT4A	This study	N/A
pEF1 V5-His A/mouse MGAT4B	This study	N/A
pEF1 V5-His A/mouse MGAT4C	This study	N/A
pEF1 V5-His A/mouse MGAT4D	This study	N/A
pEF1 V5-His A/mouse MGAT4E	This study	N/A
pEF1 V5-His A/mouse MGAT4F	This study	N/A
pEF1 V5-His A/chicken MGAT4C	This study	N/A
pEF1 V5-His A/chicken MGAT4F	This study	N/A
pcDNA-IH/chicken MGAT4F (Gln60-C-term)	This study	N/A
pEF1 V5-His A/medaka MGAT4C-rA	This study	N/A
pEF1 V5-His A/medaka MGAT4C-rB	This study	N/A
pEF1 V5-His A/medaka MGAT4D	This study	N/A
pEF1 V5-His A/mouse MGAT4D-olLec	This study	N/A
pEF1 V5-HisA/mmMGAT4D-mmLec(A)	This study	N/A
pEF1 V5-HisA/mmMGAT4D-mmLec(B)	This study	N/A
pEF1 V5-HisA/mmMGAT4D-olLec(D)	This study	N/A

**Software and algorithms**		

GraphPad Prism 8 software	GraphPad Software, Inc.	https://www.graphpad.com
BLAST	Altschul et al.^80^	https://blast.ncbi.nlm.nih.gov/Blast.cgi
MEGA 11.0.13	Tamura et al.^57^	https://www.megasoftware.net/
IQ-TREE v1.6.12	Nguyen et al.^56^; Hoang et al.^81^	http://iqtree.cibiv.univie.ac.at
MAFFT E-INS-I v	Katoh and Standley^82^	https://mafft.cbrc.jp/alignment/server/index.html
ModelFinder	Kalyaanamoorthy et al.^83^	
iTOLv7.2	Letunic and Borka^84^	https://itol.embl.de/
Synteny database	Catchen et al.^66^	Synteny Database: Select Clusters
Seq2Logo	Thomsen and Nielsen^54^	WebLogo - Create Sequence Logos
Jalview v2	Waterhouse et al.^85^	https://www.jalview.org
AlphaFold3	Abramson et al.^86^	https://github.com/google-deepmind/alphafold3
AlphaFold2	Jumper et al.^87^	https://github.com/google-deepmind/alphafold
ClustalO	Sievers and Higgins^88^	http://www.clustal.org/omega/
PyMOLv2.0	Schrodinger^89^	https://www.pymol.org/
BLASTall	Camacho et al.^90^	http://blast.ncbi.nlm.nih.gov/Blast.cgi
Cytoscape v3.10	Kohl et al.^91^	http://cytoscape.org

**Other**		

NCBI	Wheeler et al.^92^	http://www.ncbi.nlm.nih.gov
ENSEMBL	Flicek et al.^93^	http://www.ensembl.org/

### Experimental model and study participant details

#### Microbe strains

We used XL-10 Gold Ultracompetent cells (Agilent Technologies) and DH5-alpha competent cells (TAKARA) for plasmid construction.

#### Cell lines

All cell lines used in this study for cellular assays are listed in the key resources table. COS7 cells were obtained from RIKEN Cell bank, and HEK293 MGAT4A/MGAT4B DKO cells were established as described previously.^44^ All cell lines were verified to be free of mycoplasma contamination.

### Method details

#### Identification of MGAT4- (GT54) and MGAT3- (GT17) related sequences

The CAZyme predicted GT54 and GT17 proteins were identified by BLAST analysis and retrieved from public databases (e.g., NCBI and ENSEMBL). Sequences of human MGAT4 were used as a query for BLAST search of reference genomes^94^ and identify orthologues in representative metazoan taxa. The NCBI Genome Data Viewer was used to evaluate conservation splice sites junction and conserved synteny of the identified MGAT4 sequences. candidates.

#### Phylogenetic and evolutionary analyses

Phylogenetic relationships were established using the Molecular Evolutionary Genetics Analysis software version 11 (MEGA11.0.13).^57^ Protein sequences were aligned with the MUSCLE program in MEGA 11. The multiple alignments were curated manually using the Jalview program (https://www.jalview.org)^85^ to retain the informative sites. Prior to carrying out comprehensive phylogenetics, preliminary analyses were carried out for each protein family (Figure S2). Phylogenetic trees were subsequently constructed using the maximum likelihood (ML) approach and edited in Mega 11.0.13 (https://www.megasoftware.net/^57^; and graphically shaped with Adobe Illustrator CS6.

#### Synteny analysis and ancestral genomes reconstructions

Synteny analysis GT54 gene loci on a subset of vertebrate and invertebrate species was assessed by manual chromosomal walking and reciprocal BLAST searches of genes adjacent to the various MGAT4 loci in the genomes of human *Homo sapiens* (Hsap), mouse *Mus musculus* (Mmus), chicken *Gallus gallus* (Ggal), Frog *Xenopus tropicalis* (Xtro), spotted gar *Lepisosteus oculatus* (Locu), medaka *Oryzias latipes* (Olat), and salmon *Salmo salar* (Ssal) or trout *Salmo trutta* (Stru).

Paralogous blocks were also identified at the synteny database site (http://teleost.cs.uoregon.edu/synteny_db/, last accessed August 2024)^66^ (Figure S3). When a MGAT4 gene was absent in a given animal genome, we used one or two genes physically close as seed to identify the syntenic segments.

Ancestral genome reconstruction of the vertebrate and chordate ancestor in conjunction with phylogenetic and syntenic analyses was also used to rapidly assess the dynamic of MGAT4 genes evolutionary relationships, considering the reconstructions of proto-chromosomes of ancestral vertebrates (Figure S6) as previously reported for the case study of GT31 and GT29 glycosyltransferases.^51^^,^^95^^,^^96^

#### Identification of conserved motifs and structural analyses

To identify conserved motifs for each MGAT4 subfamily, MSAs were generated using the ClustalO algorithm. The number of sequences in each MSA was 36, 37, 26, 54, 7, 19, and 15 for subfamilies MGAT4A to MGAT4G, respectively. Sequence logos were generated using the Seq2Logo 2.0 program.^54^ Several corrections were applied since sequence redundancy and low sample size can affect accuracy. A pseudo-count correction was used to address the low number of sequences, with the BLOSUM62 matrix applied to adjust for the limited observations in some subfamilies. To correct sequence redundancy, clustering was performed using the Hobohm 1 algorithm, combined with a sequence-weighting method to ensure an accurate representation of conserved motifs. We computed a probability weighted Kullback-Leibler logotype that corrects the uneven distribution of amino acids, where the relative height of each amino acid symbol is proportional to the product of the probability and log-odds ratio, representing its information contribution at that position. The logo also accounts for gaps by adjusting the width of the stack accordingly.

Structural models of the globular domains of human MGAT4A (UniProt ID: Q9UM21), MGAT4C (UniProt ID Q9UBM8), and chicken MGAT4C (NCBI ID: XP_040518225.1) were built using the AlphaFold version 2.3.0 method.^87^ We generated 25 models for each protein. The first-ranked models show a high confidence pLDDT score of 92.5 for human MGAT4A, 93.4 for human MGAT4C and 93.1 for chicken MGAT4C. For overall structural presentation, the amino acid residues of mouse MGAT4A (UniProt ID: Q812G0) and mouse MGAT4C (UniProt ID: Q9D306) were retrieved and 3D structure models were generated with AlphaFold server.^86^ Structural figures were depicted with the program PyMOL (The PyMOL Molecular Graphics System, Version 2.0, Schrödinger, LLC).

#### Sequence Similarity Network

The Sequence Similarity Network (SSN) was constructed using a dataset of 218 classified sequences (numbers in parentheses indicate the sequence count for each group): MGAT4A (36), MGAT4B (37), MGAT4C (26), MGAT4D (54), MGAT4E (7), MGAT4F (19), MGAT4G (15) and invertebrates (24). Pairwise comparisons were computed using BLASTall against the custom database, with each sequence used as a query. The resulting network was visualized using Cytoscape V3.10. In this visualization, each sequence is represented as a node, with edges connecting any pair of nodes that met a predefined bit-score threshold. A force-direct layout was applied to optimize visualization of clusters and relationships within the network.

#### Molecular cloning and HEK293 cell transfection

pcDNA6 myc-His A/mouse MGAT4A,^44^ pcDNA6 myc-His A/mouse MGAT4B,^31^ pcDNA-IH/mouse MGAT4A (Ser60-C-term),^44^ and pcDNA-IH/human MGAT5 (Thr121-C-term)^79^ were constructed as described previously. pBlueScript/chicken MGAT4F was kindly provided by Dr. Naoyuki Taniguchi (Osaka International Cancer Institute).^19^ The cDNAs encoding mouse MGAT4C was amplified by PCR using the mouse brain cDNA library prepared by reverse transcription of brain total RNA derived from C57BL/6 adult male mouse as a template and cloned into pCR-Blunt II-TOPO. The cDNA fragment of mouse MGAT4C was amplified by PCR using pCR-Blunt II-TOPO construct as a template and inserted into *Hind*III/*Xho*I sites of pcDNA6 myc-His A. The cDNA encoding mouse MGAT4D was amplified by PCR using the mouse testis cDNA library prepared by reverse transcription of testis total RNA derived from C57BL/6 adult male mouse as a template and inserted into *Xho*I site in pcDNA6 myc-His A by Gibson assembly. The cDNAs encoding mouse MGAT4A, MGAT4B, MGAT4C and MGAT4D were amplified by PCR using pcDNA6 myc-His A constructs as a template and inserted into *BamH*I/*EcoR*V sites of pEF1 V5-His A by Gibson assembly. The cDNA encoding chicken MGAT4F was amplified by PCR using pBlueScript/chicken MGAT4F as a template and inserted into *BamH*I/*EcoR*V sites of pEF1 V5-His A by Gibson assembly. The cDNA encoding the luminal domain of chicken MGAT4F (Gln60-C-term) was amplified by PCR and ligated into *EcoR*I/*Xho*I sites of pcDNA-IH for the expression of N-terminally His-tagged protein. cDNAs encoding mouse MGAT4E, MGAT4F, chicken MGAT4C, and medaka MGAT4C-rA, MGAT4C-rB, MGAT4D were synthesized (Eurofins Genomics, sequences are listed in Data S1, Table S2). Using the synthesized DNAs as a template, cDNAs encoding mouse MGAT4E, MGAT4F, and chicken MGAT4C were amplified by PCR, digested with *BamH*I and *EcoR*I and inserted into *BamH*I/*EcoR*I sites of pEF1 V5-His A. Using the synthesized DNAs as a template, cDNAs encoding medaka MGAT4C-rA, MGAT4C-rB, and MGAT4D were amplified by PCR and inserted into *Bam*HI*/Eco*RI sites of pEF1 V5-His A by Gibson assembly. To construct mmMGAT4D-mmLec(A), the cDNAs encoding mouse MGAT4D (N-term to Asn415) and mouse MGAT4A lectin domain (Asp370 to C-term) were amplified by PCR using pEF1 V5-His A/mouse MGAT4A and 4D as templates and inserted into BamHI/EcoRV sites of pEF1 V5-His A by Gibson assembly. To construct mmMGAT4D-mmLec(B), the cDNAs encoding mouse MGAT4D (N-term to Asp414) and mouse MGAT4B lectin domain (Lys385 to C-term) were amplified by PCR using pEF1 V5-His A/mouse MGAT4A and 4D as templates and inserted into BamHI/EcoRV sites of pEF1 V5-His A by Gibson assembly. To construct mmMGAT4D-olLec(D), the cDNAs encoding mouse MGAT4D (N-term to Asp414) and medaka MGAT4D lectin domain (Lys378 to C-term) were amplified by PCR using pEF1 V5-His A/mouse MGAT4D and medaka MGAT4D as templates and inserted into BamHI/EcoRI sites of pEF1 V5-His A by Gibson assembly.

A MGAT4A/MGAT4B double knockout (DKO) HEK293 cell clone was established using CRISPR technique as described previously.^44^ Wild type (WT) and DKO HEK293 cells were cultured in D-MEM (high glucose) containing 10% fetal bovine serum and 50 μg/mL kanamycin under 5% CO2 conditions at 37°C. HEK cells at 70 to 90% confluency on a 10-cm dish were transfected with 10 μg of plasmids using Lipofectamine 3000 reagent (Thermo Fisher Scientific) according to the manufacturer’s protocol. Cells were collected 48–72 h after transfection and used for subsequent experiments.

#### Recombinant MGAT4 enzyme expression and enzymatic assays

HEK293 DKO cells were washed with PBS and collected by centrifugation at 400 × g for 5 min. The cells were washed again with PBS and centrifuged at 16,000 × g for 1 min. The cells were lysed with lysis buffer (50 mM Tris–HCl [pH 7.4], 150 mM NaCl, 1% Nonidet P-40, and a protease inhibitor cocktail) and sonicated. The protein concentrations of the cell lysates were measured using the Pierce BCA Protein Assay Kit (Thermo Fisher Scientific). Proteins from cell lysates were boiled in Laemmli sample buffer at 95°C for 5 min, and the proteins were resolved by 5–20% gradient SDS-PAGE. Separated proteins were transferred to nitrocellulose membranes using a semidry blotter. The membranes were blocked with 5% skim milk in Tris-buffered saline (TBS) containing 0.1% Tween 20 (TBS-T) and incubated with primary antibodies diluted with 5% skim milk in TBS-T overnight at 4°C. The anti-V5-tag antibody (mouse, clone SV5-P-K; v5ab) was from Proteintech, and the anti-GAPDH (mouse, clone 6C5; MAB374) was from Merck Millipore. After washing with TBS-T for 5 min three times, the membranes were incubated with HRP-conjugated secondary antibodies at room temperature for 1 h. Proteins were detected with the Western Lightning Plus-ECL (PerkinElmer) using the imaging system FUSION-SOLO 7s EDGE.

GnT-IV enzymatic activity toward an oligosaccharide substrate (PA-labeled biantennary *N*-glycan GnGnbi-PA) was measured using HPLC, as described previously,^73^ with slight modifications. In brief, cell lysates were incubated at 37°C in 10 μL of reaction buffer which contained 40 pmol of GnGnbi-PA, 20 mM UDP-GlcNAc, 25 mM MES (pH 7), 0.5% (v/v) Triton X-100, 5 mg/mL BSA, and 7.5 mM MnCl_2_. The reaction was stopped by boiling at 95°C for 5 min, and 40 μL of water was added to the mixture. After centrifugation at 16,000 × g for 5 min, the supernatant was analyzed by reversed-phase HPLC equipped with an ODS column (Inertsil ODS-3, GL Sciences, 4.6 × 250 mm). HPLC analysis was conducted in the isocratic mode with buffers A [20 mM ammonium acetate buffer (pH 4.0)] and B (1% 1-butanol in buffer A) mixed at a ratio of 4:1.

GnT-VI activity was measured using PA-labeled tri-antennary *N*-glycan and HPLC as follows. To prepare the acceptor substrate, soluble His-tagged human MGAT5 purified from COS7 cells^79^ was incubated in 10 μM GnGnbi-PA, 20 mM UDP-GlcNAc, 0.125 M MES (pH 6.25), 2 mg/mL BSA at 37°C for overnight. After boiling at 95 °C for 5 min and centrifugation at 16,000 × g for 5 min, the supernatant was analyzed by reversed-phase HPLC equipped with the ODS column as described above. Fractions containing the peak of the MGAT5-produced tri-antennary *N*-glycan were collected and evaporated, and the obtained glycan was dissolved with water. Cell lysates were incubated at 37°C in 10 μL of reaction buffer which contained 10 pmol of the tri-antennary acceptor, 20 mM UDP-GlcNAc, 25 mM MES (pH 7), 0.5% (v/v) Triton X-100, 5 mg/mL BSA, and 7.5 mM MnCl_2_. The reaction was stopped by boiling at 95 °C for 5 min, and 40 μL of water was added to the mixture. After centrifugation at 16,000 × g for 5 min, the supernatant was analyzed by reversed-phase HPLC equipped with the ODS column. HPLC analysis was conducted in the isocratic mode with buffers A [20 mM ammonium acetate buffer (pH 4.0)] and B (1% 1-butanol in buffer A) mixed at a ratio of 19:1.

#### MS analysis of enzymatic product

Products of mouse MGAT4A, MGAT4C, chicken MGAT4C, MGAT4F, and medaka MGAT4C-rA, MGAT4D were purified as follows. Soluble His-tagged enzymes (mouse MGAT4A and chicken MGAT4F) were expressed and purified from COS7 cells through Ni-column as described previously.^44^ For mouse MGAT4C, chicken MGAT4C, medaka MGAT4C-rA and MGAT4D, cell lysates expressing these enzymes were used as enzyme source. MGAT4A and MGAT4D were incubated at 37°C in a buffer containing 25 mM MES (pH 7), 0.5% (v/v) Triton X-100, 5 mg/mL BSA, 7.5 mM MnCl_2_, 82.3 μM GnGnbi-PA, and 20 mM UDP-GlcNAc. MGAT4C, MGAT4C-rA, and MGAT4F were incubated at 37°C in a buffer containing 25 mM MES (pH 7), 5 mg/mL BSA, 7.5 mM MnCl_2_, 10 μM tri-antennary acceptor, and 20 mM UDP-GlcNAc. The reactions were stopped by boiling at 95 °C for 5 min. After centrifugation at 16,000 × g for 5 min, the supernatant was analyzed by reversed-phase HPLC as described above, and fractions containing the enzyme products were collected and evaporated. The fractions were solubilized with water and mixed with a matrix solution (10 mg/mL 2,5-dihydroxybenzoic acid in 50% acetonitrile) at a 1:1 ratio. The mixture was then spotted onto a target plate and analyzed by MALDI-TOF-MS using an Autoflex Speed or UltrafleXtreme MALDI-TOF/TOF mass spectrometer (Bruker Daltonics) in positive-ion reflector mode. Acquired spectra were processed using flexAnalysis 3.4 software (Bruker Daltonics).

## Supplemental information

Document S1. Figures S1–S9

Table S1. List of MGAT4 sequences, names, accession number, and species used in this study

Table S2. Sequences of primers and cDNA used for the construction of expression vectors
