# Supplementary material for: Evolutionary analyses of the animal glycosyltransferase family 54 reveals two β1,4-N-acetylglucosaminyltransferase families
Source: iScience. 2025 Oct 15;28(11):113788. doi: 10.1016/j.isci.2025.113788 (PMC12616123; doi:10.1016/j.isci.2025.113788)
Supplement: Document S1. Figures S1–S9 [file mmc1.pdf]

iScience, Volume *28*

Supplemental information

Evolutionary analyses of the animal glycosyltransferase family 54 reveals two β1,4-N-acetylglucosaminyltransferase families

Aoi Morigo, Roxana Elin Teppa, Masamichi Nagae, Hirokazu Yagi, Yasuhiko Kizuka, and Anne Harduin-Lepers

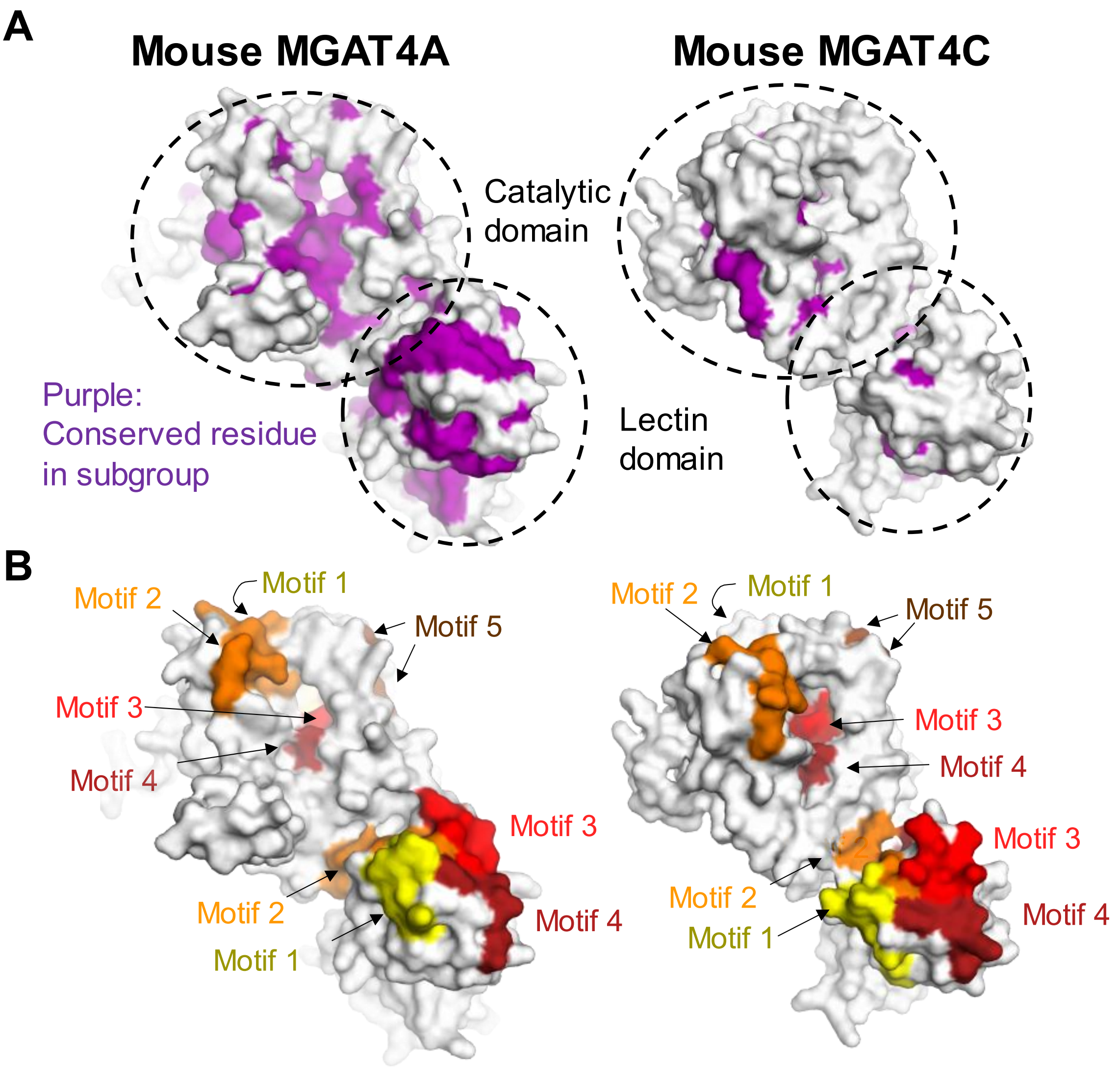

Supplemental Figure S1: Overall structure of mouse MGAT4 enzymes.

The overall structures of luminal regions of mouse MGAT4A and MGAT4C (predicted by AlphaFold3) are shown. A) Residues conserved in each subgroup (mouse MGAT4A, MGAT4B, MGAT4D for left and mouse MGAT4C, MGAT4E, MGAT4F for right) are colored in purple. B) The conserved motifs shown in Figure 3 and 4 are mapped (motif 1: yellow, motif 2: orange, motif 3: red, motif 4: dark red and motif 5: brown).

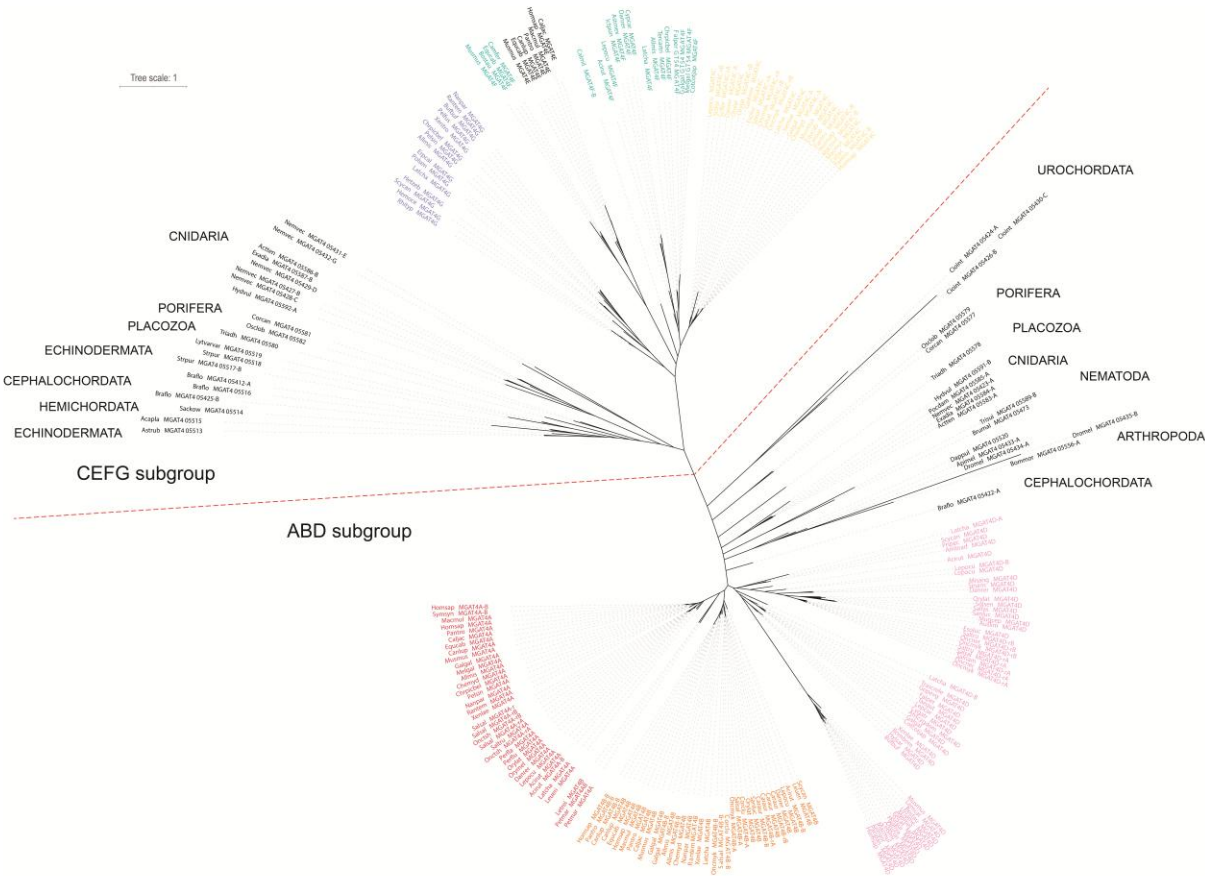

Supplemental Figure S2: Phylogenetic Maximum likelihood tree obtained with IQ-tree showing evolutionary relationships between metazoan GT54 sequences and sister subgroups ABD and CEFG.

The construction of the phylogenetic tree was carried out using the IQ-TREE (1.6.12) software, using the maximum likelihood method and performing ultrafast bootstrap (UFBoot) analysis with 1000 replicates [1]. To select the best-fitting model of molecular evolution, we used the proposed model tool ModelFinder in the program IQ-Tree v1.6.12 [2], which selected JTT+F+R7 (Best-fit model according to BIC). The 232 GT54 sequences (51 invertebrate and 181 vertebrate sequences with 1029 amino acid sites) were aligned using the software MAFFT v.7 [3], allowing the program to choose the alignment strategy (L-INS-i). The number of constant sites was 273 (= 26.5306% of all sites). The scale denotes substitutions per site and colors represent the seven vertebrate GT54 clades (the color code is the one used in Figure 5). The tree was modified using the software iTOL (version 6.9) [4].

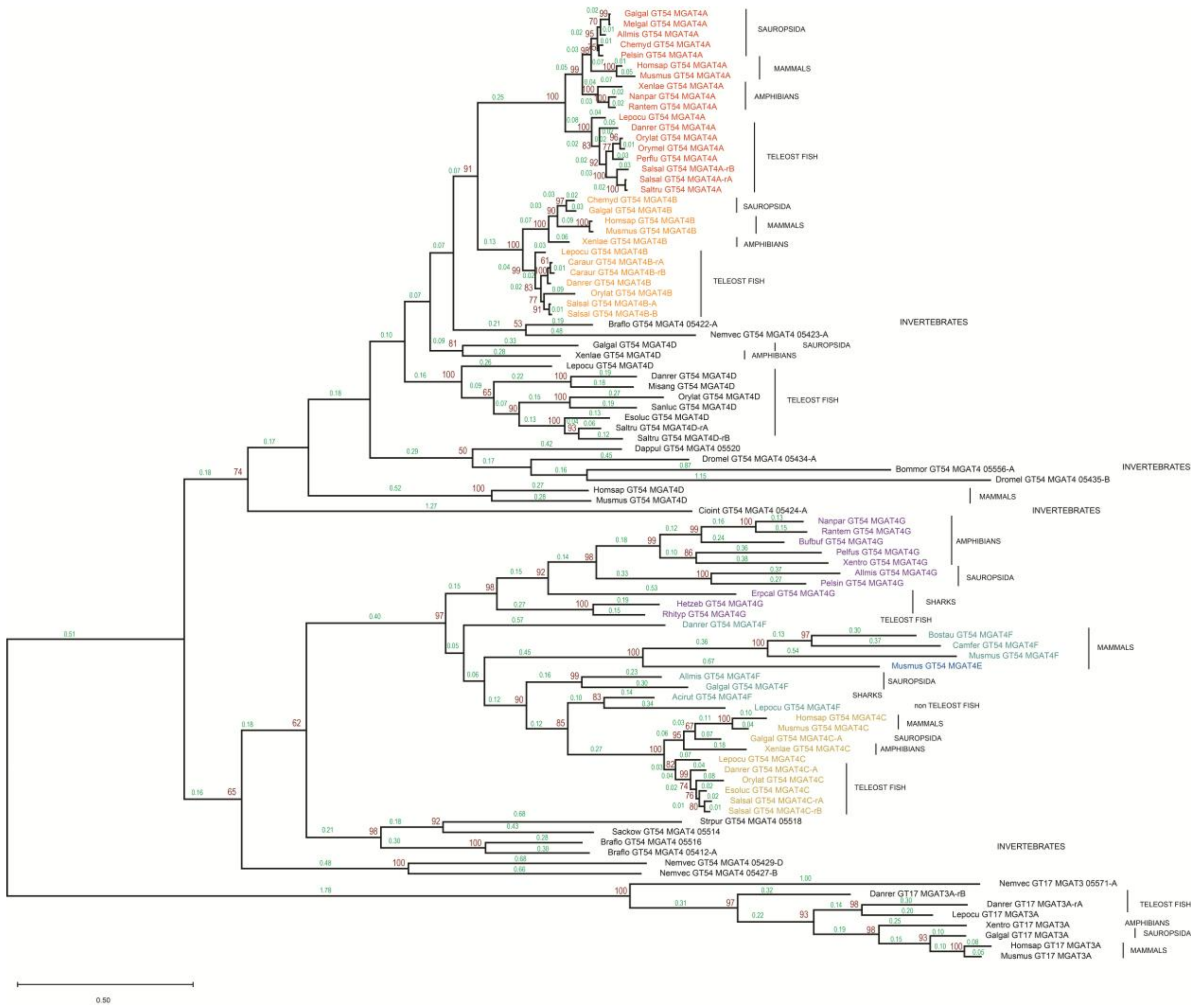

Supplemental Figure S3: Maximum Likelihood phylogenetic tree corresponding to Figure 5A with statistical analysis

The evolutionary history of selected MGAT4 sequences was inferred by using the Maximum Likelihood method and JTT matrix-based model [5]. The tree with the highest log likelihood (-55845.83) is shown. Initial tree(s) for the heuristic search were obtained automatically by applying Neighbor-Join and BioNJ algorithms to a matrix of pairwise distances estimated using the JTT model, and then selecting the topology with superior log likelihood value. The tree is drawn to scale, with branch lengths measured in the number of substitutions per site (next to the branches). This analysis involved 92 amino acid sequences, both from vertebrate and invertebrate species. There was a total of 800 positions in the final dataset. Evolutionary analyses were conducted in MEGA11[6].

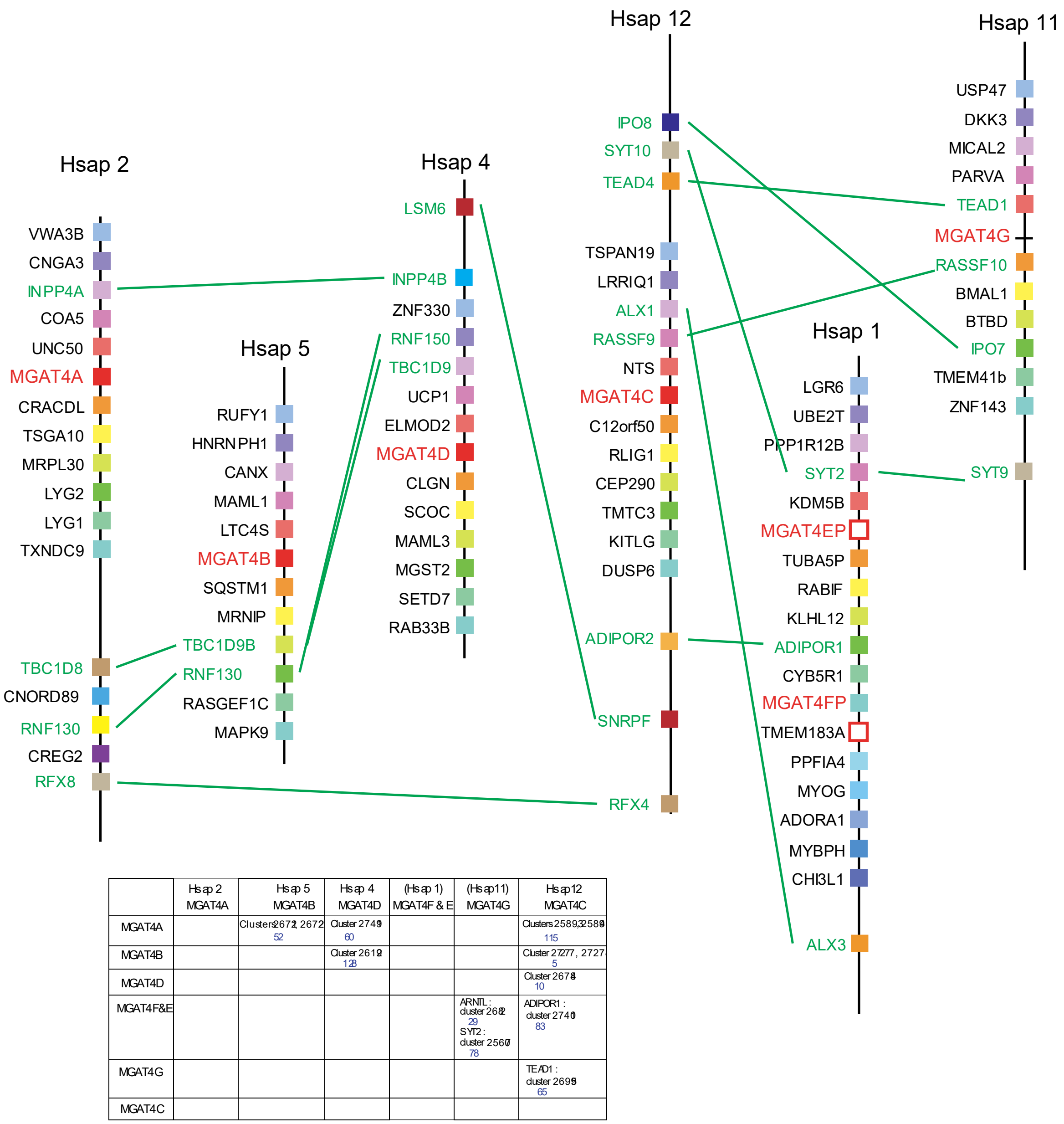

	Hsap 2 MGAT4A	Hsap 5 MGAT4B	Hsap 4 MGAT4D	(Hsap 1) MGAT4F & E	(Hsap11) MGAT4G	Hsap12 MGAT4C
MGAT4A		Clusters 2672, 2672 52	Cluster 2749 60			Clusters 2589, 2589 115
MGAT4B			Cluster 2612 128			Cluster 27277, 2727[illegible] 5
MGAT4D						Cluster 2678 10
MGAT4F&E					ARNTL : cluster 2682 29 SYT2 : cluster 2567 78	ADIPOR1 : cluster 2740 83
MGAT4G						TEAD1 : cluster 2695 65
MGAT4C						

Supplemental Figure S4: Synteny and paralogy analyses in the human genome.

The homologous MGAT4 genes were localized on the human chromosome (Hsap) and their number are indicated above each chromosomal segment illustrated here. Genes are indicated by colored squares, pseudogenes by white squares delineated by red color and lost genes are indicated by a hyphen. Genes indicated in green have paralogs on at least two human chromosomes. The human MGAT4 genes are indicated in red. The table below indicates the number of human paralogues identified from the synteny database at Uoregon Site (http://syntenydb.uoregon.edu/synteny_db/) last accessed July 2024 [7].

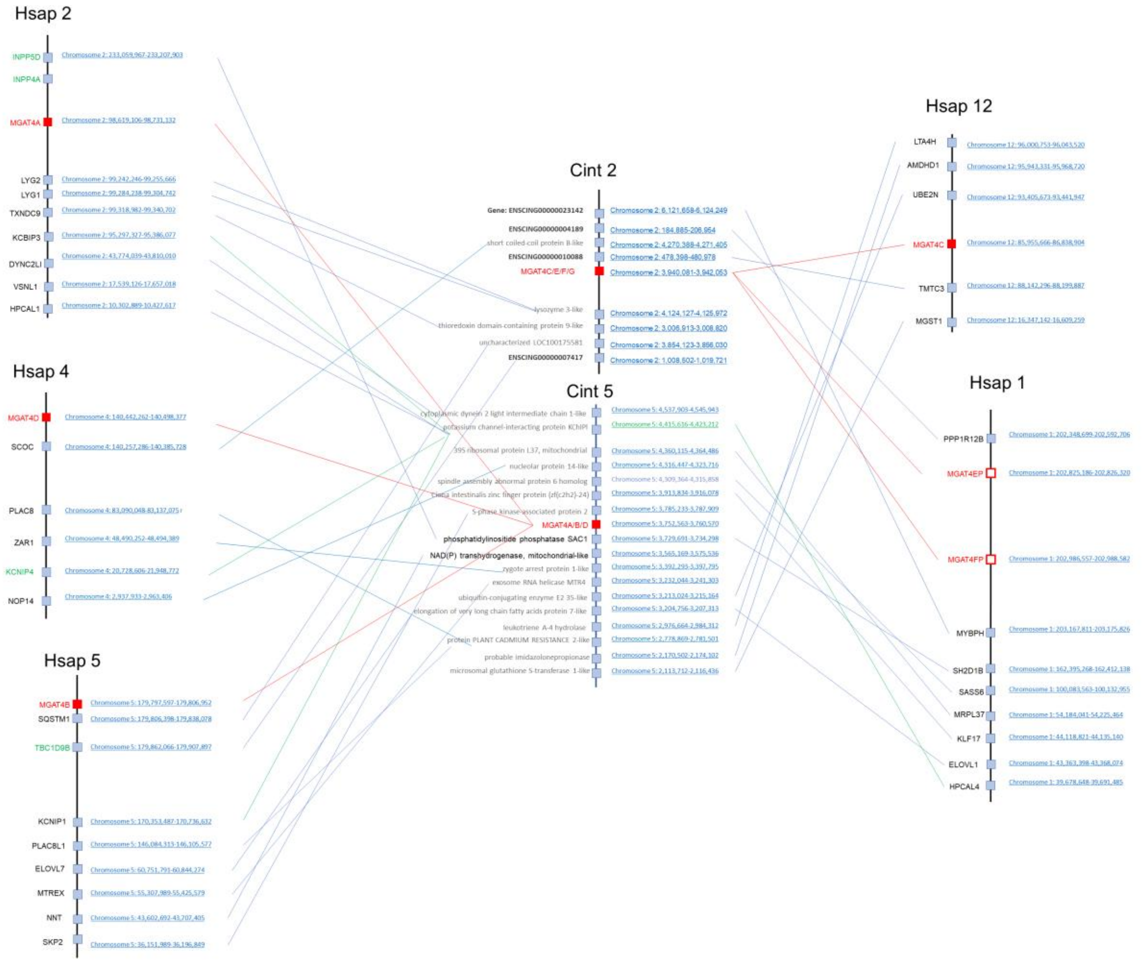

Supplemental Figure S5: Synteny analyses in the human (Hsap) and *Ciona intestinalis* (Cint) genomes.

This figure depicts the conserved synteny between the chromosomal regions hosting two *Ciona intestinalis* MGAT4 homologues (MGAT4A/B/D and MGAT4C/E/F/G) indicated in red squares on the *C. intestinalis* chromosomes Cint 2 and Cint 5 and five regions hosting human MGAT4 homologues MGAT4A, MGAT4B, MGAT4D, MGAT4C, MGAT4EP and MGAT4EF on human chromosomes (Hsap) 2, 4, 5, 12 and 1. Each *C. intestinalis* gene is shown together with its corresponding closest human orthologue(s). Synteny between the human MGAT4 loci and related gene on Cint 2 and Cint 5 was assessed by chromosomal walking and reciprocal TBLASTN searches of genes adjacent to MGAT4 loci in human and Ciona genome databases.

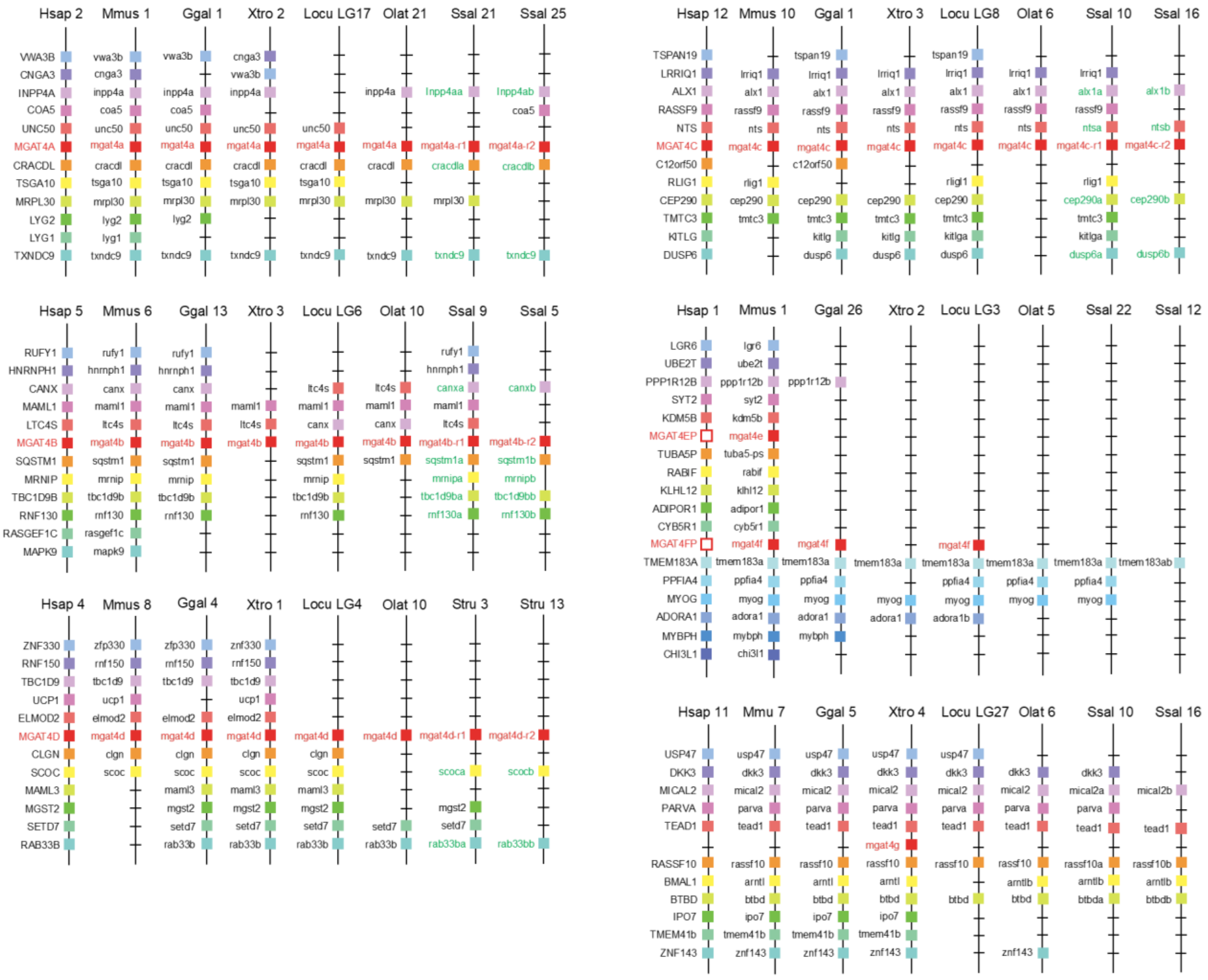

Supplemental Figure S6: Synteny analyses around the vertebrate MGAT4 genes loci. The physically-mapped genomes of human *Homo sapiens* (Hsap), mouse *Mus musculus* (Mmus), chicken *Gallus gallus* (Ggal), Frog *Xenopus tropicalis* (Xtro), spotted gar *Lepisosteus oculatus* (Locu), medaka *Oryzias latipes* (Olat), and salmon *Salmo salar* (Ssal) or trout *Salmo trutta* (Stru) were used to identify conserved gene neighbors of the MGAT4 genes. Paralogous genes located upstream and downstream each MGAT4 locus was searched using BLAST at the NCBI database and the latest ENSEMBL data set (ENS70). Each diagram represents the order of genes on the chromosome in the vicinity of the relevant MGAT4 genes. The names of MGAT4 genes are red. Paralogous genes found in salmonids are indicated in green. Synteny is highly conserved in the first group of MGAT4 A, MGAT4B and MGAT4D represented on the left side. In the second group represented on the right side, human MGAT4E and MGAT4F are pseudogenes and are indicated as red open squares. MGAT4E gene is only present in mammals and likely results from a translocation in the mammals' ancestor. MGAT4G was only found in amphibians, squamates and sharks and likely disappeared in teleost fish and mammals.

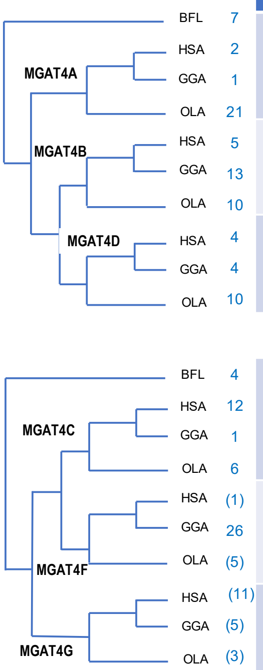

Block ID	Gnathost. Anc. (GNA) (Putnam 2008)	Teleost Anc. Chrom. (Kasahara 2007)	Chord. Link. Gr. (CLG) (Putnam 2008; Simakov 2020)	Verteb. Anc. Chrom. (VAC) (Nakatani 2007)
11	unk	c	F	unk
28	C2	g	F	C
23 25 24	C1	f f l	F	C
64 66 68	D0	k k k	E/O	D
3	D2	l	E/O	D
62 60	D1	j j	E/O	D

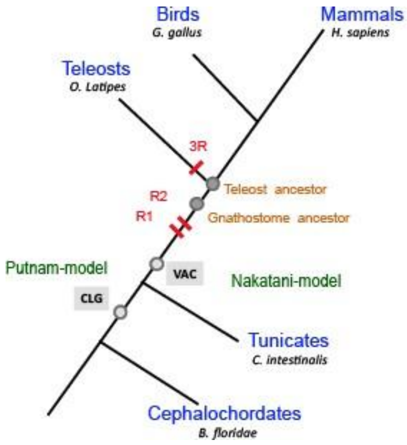

Supplemental Figure S7: Reconstruction of ancestral genome to assess the vertebrate MGAT4 subfamilies' origin.

A simplified phylogenetic tree is represented on the right side that illustrates evolution of chordate genomes with ancestral genome reconstruction in the ancestor of vertebrates. This approach is based on the N-model of Nakatani et al.[8] and P-model of Putnam et al. [9,10]. The 2R-duplicated genes are found on one of the 10 vertebrate ancestral chromosomes (VAC) in the pre-2R genome and designated A–J. Similarly, they are found on one of the 17 hypothetical ancestral chordate linkage groups (CLG) in the pre-2R genome numbered A to Q in the P-model [9,10] and on one of the 13 teleost ancestral proto-chromosomes named a to m in the pre-3R genome reconstructed from the extant fish genomes [11]. After the two WGD-2R events, they are found on one of the four linkage groups with shared synteny (for instance, Gnathostome ancestor (GNA) proto-chromosomes A0, A1, A2 and A3). Conserved synteny was established for MGAT4A, MGAT4B and MGAT4D and surrounding gene loci and the blocks associated to these genes corresponded to the CLG proto-chromosome F in the P-model and to the ancestral proto-vertebrate chromosome CLG-C in the N-model located on GNA proto-chromosomes C1 and C2 (it could not be assigned for MGAT4A). Conserved synteny was established for MGAT4C, MGAT4F, and MGAT4G and surrounding gene loci and the blocks associated to these genes corresponded to the CLG proto-chromosome E/O in the P-model and to the ancestral proto-vertebrate chromosome CLG-D in the N-model located on GNA proto-chromosomes D0, D2 and D1.

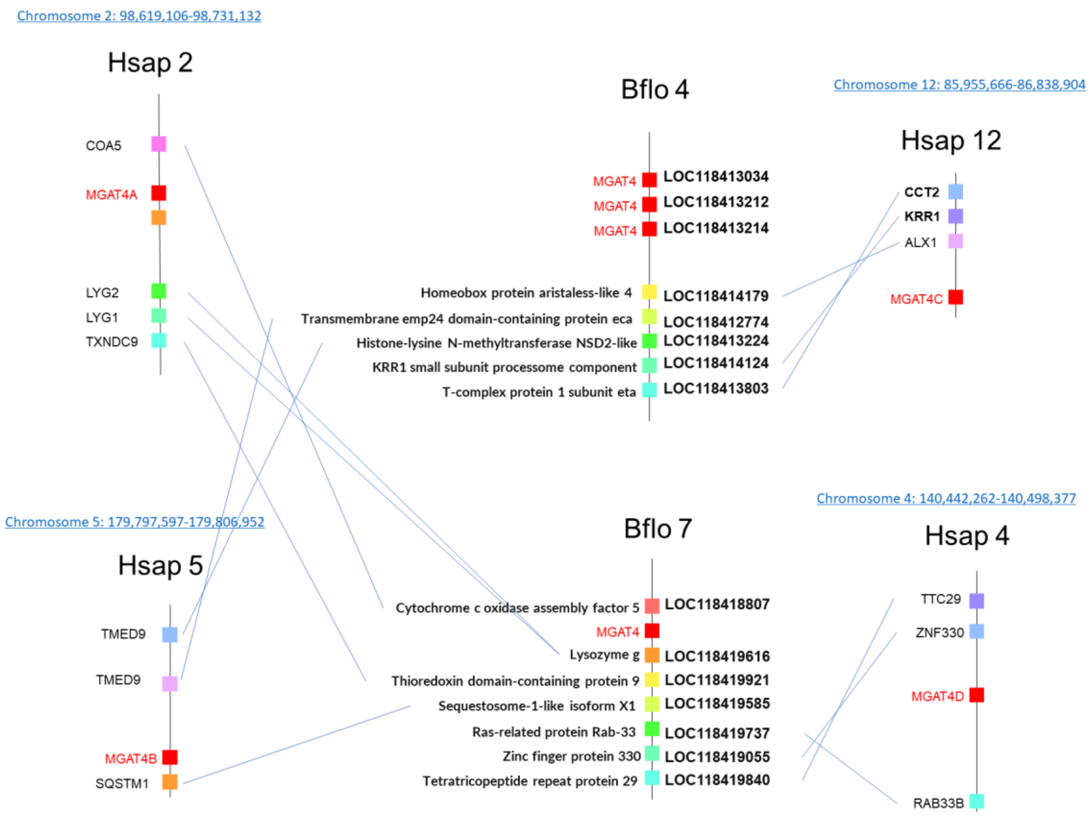

Supplemental Figure S8: Synteny analysis in the human and *Branchiostoma floridae* genomes.

This figure depicts the conserved synteny between the chromosomal regions hosting *Branchiostoma floridae* MGAT4 homologues (MGAT4A/B/D and MGAT4C/E/F/G) indicated in red squares on the *B. floridae* chromosomes Bflo 4 and Bflo 7 and four regions hosting human MGAT4 homologues MGAT4A, MGAT4B, MGAT4D and MGAT4C on human chromosomes (Hsap) 2, 4, 5 and 12. Each *B. floridae* gene is shown together with its corresponding closest human orthologue(s). Synteny between the human MGAT4 loci and related gene on Bflo 4 and Bflo 7 was assessed by chromosomal walking and reciprocal TBLASTN searches of genes adjacent to MGAT4 loci in human and amphioxus genome databases.

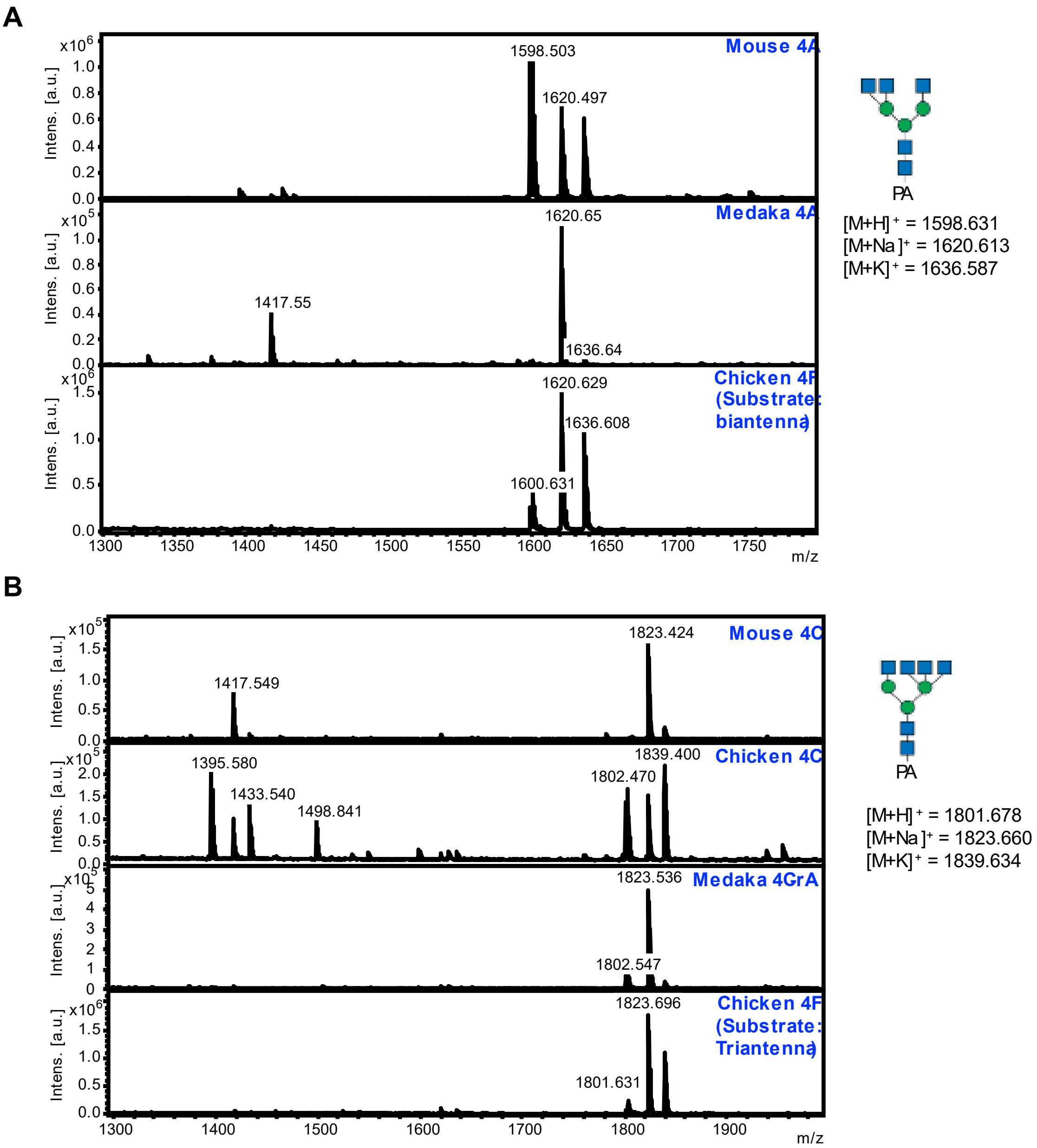

Supplemental Figure S9: MALDI-TOF-MS profiles of reaction products catalyzed by individual GlcNAc-transferases.

MS spectra of the MGAT4 enzyme products obtained using bi-antennary (A) and tri-antennary (B) glycans as substrates. Peaks corresponding to protonated, sodium-adducted, and/or potassium-adducted species are observed.

References

1. Hoang, D.T., Chernomor, O., Von Haeseler, A., Minh, B.Q., and Vinh, L.S. (2018). UFBoot2: improving the ultrafast bootstrap approximation. Molecular biology and evolution *35*, 518-522.
2. Trifinopoulos, J., Nguyen, L.-T., von Haeseler, A., and Minh, B.Q. (2016). W-IQ-TREE: a fast online phylogenetic tool for maximum likelihood analysis. Nucleic acids research *44*, W232-W235.
3. Katoh, K., and Standley, D.M. (2013). MAFFT multiple sequence alignment software version 7: improvements in performance and usability. Molecular biology and evolution *30*, 772-780.
4. Letunic, I., and Bork, P. (2024). Interactive Tree of Life (iTOL) v6: recent updates to the phylogenetic tree display and annotation tool. Nucleic Acids Research *52*, W78-W82. 10.1093/nar/gkae268.
5. Jones, D.T., Taylor, W.R., and Thornton, J.M. (1992). The rapid generation of mutation data matrices from protein sequences. Bioinformatics *8*, 275-282.
6. Tamura, K., Stecher, G., and Kumar, S. (2021). MEGA11: Molecular Evolutionary Genetics Analysis Version 11. Molecular Biology and Evolution *38*, 3022-3027. 10.1093/molbev/msab120.
7. Catchen, J.M., Conery, J.S., and Postlethwait, J.H. (2009). Automated identification of conserved synteny after whole-genome duplication. Genome Res *19*, 1497-1505.
8. Nakatani, Y., Takeda, H., Kohara, Y., and Morishita, S. (2007). Reconstruction of the vertebrate ancestral genome reveals dynamic genome reorganization in early vertebrates. Genome Res *17*, 1254-1265. 10.1101/gr.6316407.
9. Putnam, N.H., Butts, T., Ferrier, D.E., Furlong, R.F., Hellsten, U., Kawashima, T., Robinson-Rechavi, M., Shoguchi, E., Terry, A., Yu, J.K., et al. (2008). The amphioxus genome and the evolution of the chordate karyotype. Nature *453*, 1064-1071. 10.1038/nature06967.
10. Simakov, O., Marlétaz, F., Yue, J.-X., O'Connell, B., Jenkins, J., Brandt, A., Calef, R., Tung, C.-H., Huang, T.-K., Schmutz, J., et al. (2020). Deeply conserved synteny resolves early events in vertebrate evolution. Nature ecology & evolution *4*, 820-830. 10.1038/s41559-020-1156-z.
11. Kasahara, M., Naruse, K., Sasaki, S., Nakatani, Y., Qu, W., Ahsan, B., Yamada, T., Nagayasu, Y., Doi, K., Kasai, Y., et al. (2007). The medaka draft genome and insights into vertebrate genome evolution. Nature *447*, 714-719. 10.1038/nature05846.
